# Huqi formula suppresses hepatocellular carcinoma growth by modulating the PI3K/AKT/mTOR pathway and promoting T cell infiltration

**DOI:** 10.1186/s13020-025-01061-w

**Published:** 2025-02-19

**Authors:** Donghao Yin, Xiang Li, Xuemeng Yang, Xiaofei Shang, Zhen Li, Jiahao Geng, Yanyu Xu, Zijing Xu, Zixuan Wang, Zimeng Shang, Zhiyun Yang, Linlan Hu, Quanwei Li, Jiabo Wang, Xinhua Song, Xiuhui Li, Xiaojun Wang

**Affiliations:** 1 Beijing Youan Hospital, Capital Medical University, Beijing, 100069 China; 2 Department of Natural Medicines, School of Traditional Chinese Medicine, Laboratory for Clinical Medicine, Capital Medical University, Beijing, 100069 China; 3 Key Laboratory of New Animal Drug Project, Gansu Province, Key Laboratory of Veterinary, Pharmaceutical Development of Ministry of Agriculture, Lanzhou Institute of Husbandry and Pharmaceutical Sciences, Chinese Academy of Agricultural Sciences, Lanzhou, 730050 China; 4 Cao Xian People’s Hospital, Shandong, 274400 China; 5 Laboratory for Clinical Medicine, Center for Integrative Medicine, Beijing Ditan Hospital, Capital Medical University, No. 8 Jingshun East Street, Beijing, 100015 People’s Republic of China

**Keywords:** Hepatocellular carcinoma, Huqi formula, Apoptosis, T cells, Immunotherapy

## Abstract

**Background:**

Hepatocellular carcinoma (HCC) poses ongoing difficulties for public health systems due to its high incidence and poor prognosis. Huqi formula (HQF), a well-known prescription in traditional Chinese medicine, has demonstrated notable clinical effectiveness in the treatment of HCC. However, the mechanisms underlying its therapeutic effects have yet to be completely elucidated.

**Purpose:**

This study aimed to investigate the anti-HCC effects of HQF and its underlying mechanisms.

**Methods:**

Chemical profiling and quantification of HQF were conducted by LC–MS and HPLC. Orthotopic and subcutaneous tumor models were established through hydrodynamic injection of Akt/Nras plasmids and subcutaneous injection of c-Met/sgPten cells, respectively, to evaluate the therapeutic effects of HQF on HCC. Network pharmacology, RNA-Seq, molecular docking, Western blot, and flow cytometry were employed to assess the anti-HCC mechanisms.

**Results:**

LC–MS analysis identified 41 components, with HPLC quantification showing salvianolic acid B as the most abundant compound (0.303%). In Akt/Nras and c-Met/sgPten-induced HCC models, HQF significantly reduced tissue damage, improved liver function, and inhibited HCC progression. Mechanistic studies revealed that HQF induced apoptosis in HCC cells by downregulating p-PI3K, p-AKT, and p-mTOR expression, with molecular docking indicating the strongest binding affinity between salvianolic acid B and PI3K. HQF further enhanced CD4^+^ and CD8^+^ T cell infiltration within the tumor microenvironment. When combined with PD-1 therapy, HQF improved therapeutic efficacy against HCC. Finally, toxicity assays confirmed the safety profile of HQF.

**Conclusion:**

HQF demonstrated significant anti-HCC effects and a synergistic effect with PD-1, could be used as an alternative therapeutic agent for HCC.

**Graphical Abstract:**

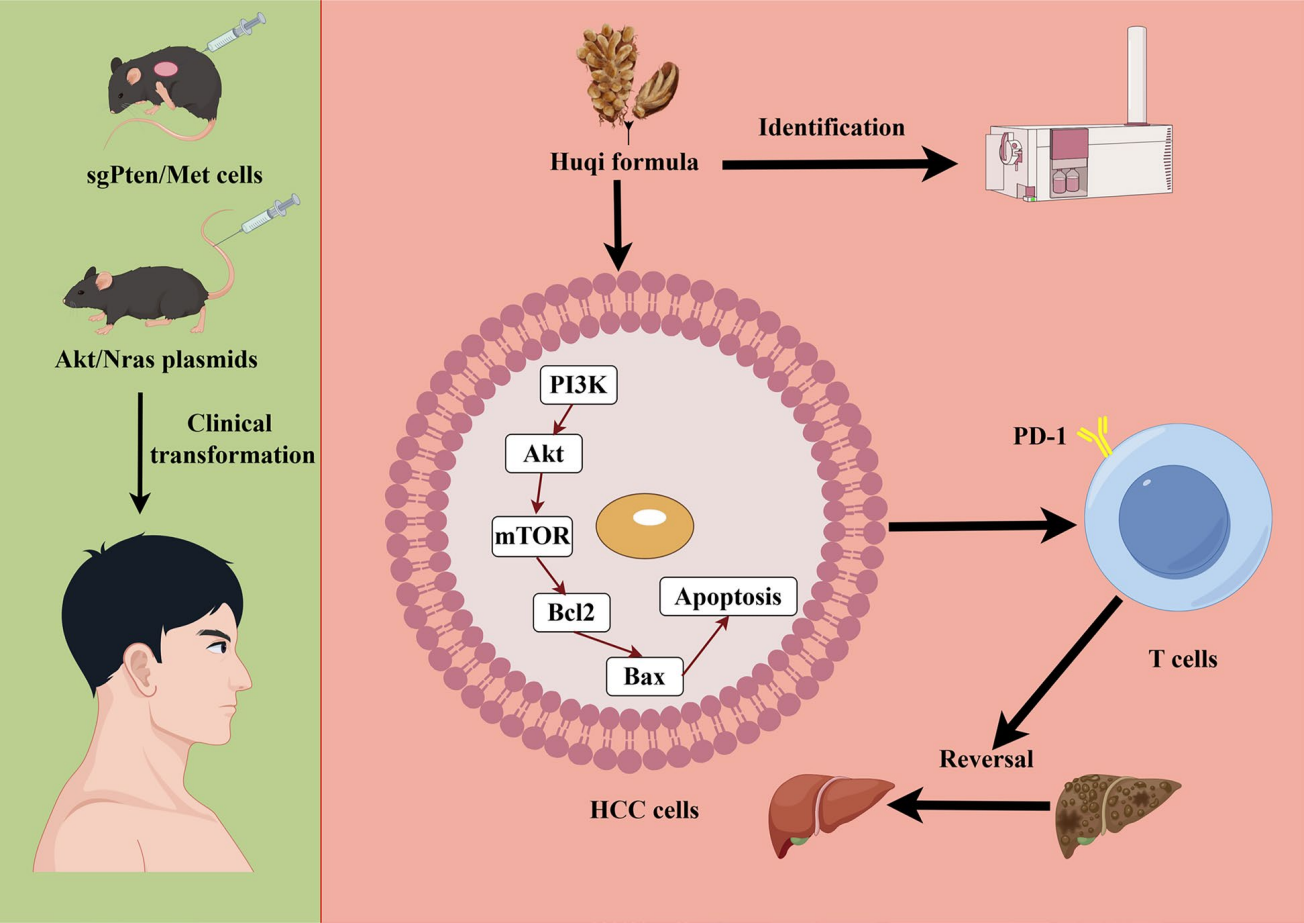

**Supplementary Information:**

The online version contains supplementary material available at 10.1186/s13020-025-01061-w.

## Introduction

Globally, hepatocellular carcinoma (HCC) poses a major challenge to healthcare systems due to its high prevalence and significant mortality rate [1]. Recent global cancer statistics reveal a steady rise in HCC incidence, particularly in regions such as Asia and Africa [2, 3]. While surgery, radiotherapy, chemotherapy, and targeted therapies are commonly used for HCC treatment, these options often result in significant side effects, and the prognosis remains poor due to challenges in early detection and high recurrence rates [4]. This indicates an urgent need for developing therapies with better efficacy and fewer side effects.

Traditional Chinese medicine (TCM) has recently attracted increasing focus for its ability to combat diseases such as HCC. Unlike conventional therapies, TCM provides therapeutic advantages with fewer side effects [5, 6]. Extensive research has validated the efficacy and safety of TCM in managing HCC [7, 8]. Huqi formula (HQF), developed by professor Ying Qian from extensive clinical experience, is a modified formula based on the traditional TCM prescriptions Yiguanjian and Biejiajianwan [9]. The final decoction includes *Viscum coloratum*, *Astragalus membranaceus*, *Salvia miltiorrhiza*, *Oldenlandia diffusa*, *Curcuma phaeocaulis*, and *Phyllanthus urinaria* [10]. HQF has been widely used at Beijing YouAn Hospital, affiliated with Capital Medical University, in treating HCC patients. Clinical studies have shown that it significantly improves TCM syndrome scores and enhances the quality of life in HCC patients [11]. However, the mechanism of this decoction has not yet been elucidated.

The progression of HCC is driven by the dysregulation of key oncogenic pathways, such as the phosphoinositide 3-kinase/protein kinase B/mammalian target of rapamycin (PI3K/AKT/mTOR) signaling pathway, which is known to promote cell proliferation, inhibit apoptosis, and enhance cancer cell survival [12]. Due to its critical role in driving tumor growth and resistance to conventional treatments, this pathway has garnered significant attention as a therapeutic target. AKT reduces pro-apoptotic proteins such as Bax while increasing anti-apoptotic proteins like Bcl-2, thereby decreasing mitochondrial permeability and inhibiting apoptosis [13, 14]. Inhibiting this pathway can restore apoptotic signaling and suppress tumor growth.

In this study, an orthotopic HCC model (via hydrodynamic injection of Akt/Nras plasmids) and a subcutaneous tumor model (using c-Met/sgPten cells) were established to evaluate the therapeutic effects of HQF on HCC. Phytochemical analysis, network pharmacology, RNA-seq, molecular docking, and molecular biology experiments were employed to investigate the mechanisms by which HQF combats HCC.

## Materials and methods

### Reagents and antibodies

Medicinal herbs obtained from Beijing Tong Ren Tang Pharmacy include *Viscum coloratum* (Kom.) Nakai (30 g), *Astragalus membranaceus* (Fisch.) Bunge (30 g), *Salvia miltiorrhiza* Bunge (15 g), *Curcuma phaeocaulis* Valeton (9 g), *Oldenlandia diffusa* (Willd.) Roxb. (30 g), and *Phyllanthus urinaria* L. (15 g). The PLC5/PRF/5 cell line was obtained from Pricella Biotechnology Co., Ltd. (Wuhan, Hubei), and the SNU449 cell line was obtained from Meisen Cell Technology Co., Ltd. (Zhejiang, China), both of which were cultured under standard conditions. Specifically, the cells were maintained in Dulbecco’s Modified Eagle Medium (DMEM) (for PLC/PRF/5) or RPMI-1640 medium (for SNU449) supplemented with 10% fetal bovine serum (FBS), 100 U/mL penicillin, and 100 µg/mL streptomycin. The cells were incubated at 37 °C in a humidified atmosphere containing 5% CO₂. Media were replaced every 2–3 days, and cells were passaged when reaching 70–80% confluence using 0.25% trypsin–EDTA for detachment. The VAHTS Universal V6 RNA-seq Library Prep Kit for Illumina was used for RNA-seq library preparation. The Annexin V-FITC/PI dual staining kit was purchased from BD Bioscience (USA), and the BeyoClick™ EdU-488 assay kit was purchased from Beyotime (Shanghai, China). The following antibodies were purchased from eBioscience™: CD8a, F4/80, Ly-6G, CD3, PD-1, Fixable Viability Dye, Ly-6G/Ly-6C, CD86, CD25, CD11b, CD206, CTLA-4, FOXP3, NK1.1, CD49b, and CD4. The antibodies Bax, cleaved caspase-3, PI3K, AKT, P-AKT, mTOR, and P-mTOR were purchased from Cell Signaling Technology (USA). The antibodies CD8, CD31, and CD34 were purchased from Abcam. The antibodies PCNA and P-PI3K were purchased from Affinity (Melbourne, Australia). The antibodies Ki-67 and Bcl-2 were purchased from Proteintech (Wuhan, China).

### Preparation of HQF

The herbs were first soaked in purified water at a 1:10 ratio for 30 min, then simmered for 1 h to complete the decoction. After filtering the mixture to eliminate solid residues, the liquid was cooled to room temperature and stored at 4 °C. The solid residues were steeped again in water at eight times their weight, and the decoction was repeated. The filtrates from both extractions were combined, concentrated using rotary evaporation, and vacuum-dried at 60 °C to produce HQF crude powder [15]. Each dose of HQF contained 129 g of raw medicinal materials. After the extraction process described above, 15.78 g of HQF crude powder was obtained, resulting in an extraction yield of 12.23%.

### Liquid chromatography-time of flight-mass spectrometry (LC-TOF–MS) analysis

An Agilent 120 EC-C18 RP-HPLC system, integrated with an Agilent 6550 iFunnel mass spectrometer (TripleTOF 6600, ESI), was employed for the experiment. Ionization was performed in both modes, with TOF–MS and TOF–MS/MS scanning *m/z* ranges of 100–1200 and 50–1200, respectively. The system operated with GS1 at 50 psi, GS2 at 55 psi, CUR at 35 psi, and a temperature of 500 °C. The ion source voltages were set to − 4500 V and + 5500 V, with DP and CE values of − 80 V/ + 80 V and − 10 V/ + 10 V, respectively. For MS2, DP remained at − 80 V/ + 80 V, with CE adjusted to − 40 V/ + 40 V. Chromatographic separation was achieved using an Agilent EC-C_18_ column (4.6 × 150 mm, 2.7 μm) in a mobile phase of 0.1% formic acid (A) and acetonitrile (B). Gradient elution increased from 8 to 90% B over 17 min, with a flow rate of 0.3 mL/min, and a column temperature held at 35 °C.

### *Quantification of seven compounds in HQF *via* high-performance liquid chromatography (HPLC)*

The chemical composition and concentration of key compounds in HQF aqueous extract were determined using a Waters 2695 HPLC system, equipped with a Waters Symmetry C18 column (4.6 × 150 mm, 5 μm). The mobile phase comprised acetonitrile (A) and 0.1% formic acid in water (B) [16]. Operating at a flow rate of 1 mL/min and a column temperature of 35 °C, detection was performed at 280 nm and 350 nm.

### The effects of HQF on c-Met/sgPten-induced subcutaneous HCC model

Eighteen male C57BL/6 J mice, 4 weeks old, were acquired from Vital River Laboratory Animal Technology Co., Ltd (Beijing, China). This study was conducted with approval from the Animal Ethics Committee of Capital Medical University (AEEI-2021–018). Primary HCC cells (c-Met/sgPten) were extracted from an c-Met/sgPten/SB HCC mouse model and subcutaneously injected (2.5 × 10^5^ cells/mL) into the right flank of each mouse. Five days later, the mice were randomly assigned to one of three groups: control (Saline), HQF high-dose (33.54 g/kg/day, n = 6), and HQF low-dose (16.77 g/kg/day, n = 6), with treatment continuing for 22 days. Tumor size was recorded every 3 days using calipers. At the end of the study, tissues were collected for hematoxylin and eosin (HE) staining, TUNEL, and immunohistochemical staining to assess pathological changes in HCC tissues.

### The effects of HQF on an Akt/Nras/SB-driven orthotopic HCC model

An Akt/Nras/SB-driven HCC model was generated in twenty-one 5-week-old male C57BL/6 J mice using hydrodynamic injection of Akt/Nras/SB plasmids via the tail vein [17]. Tumor formation was confirmed after one week. In the second week, mice were randomly divided into control (Saline), HQF high-dose (33.54 g/kg/day, n = 7), and HQF low-dose (16.77 g/kg/day, n = 7) groups. Treatment lasted for four weeks with daily administration. Baseline tumor imaging was conducted before treatment using an in vivo imaging system. Upon completion of the treatment, liver and other organs were harvested for HE staining and immunohistochemistry to evaluate the impact of HQF on tumor progression.

### Immunohistochemistry

Tumor tissues were harvested, fixed in 4% paraformaldehyde for 24 h, and embedded in paraffin. Tissue Sects. (4 μm thick) were deparaffinized in xylene and rehydrated through graded ethanol. Antigen retrieval was performed by heating the sections in citrate buffer (pH 6.0) at 95 °C for 15 min. After cooling to room temperature, the sections were washed in PBS and treated with 3% hydrogen peroxide for 10 min to block endogenous peroxidase activity. Non-specific binding was blocked by incubating the sections with 5% BSA for 30 min at 25 °C. The sections were then incubated overnight at 4 °C with primary antibodies. After washing with PBS, the sections were incubated with an HRP-conjugated secondary antibody at room temperature for 1 h. DAB (3,3′-diaminobenzidine) substrate solution was used for visualization, followed by counterstaining with hematoxylin. The slides were then dehydrated through graded ethanol, cleared in xylene, and mounted with neutral balsam.

### Effects of HQF on cell viability, colony formation, and migration in PLC/PRF/5 and SNU449 cells

After 48 h of HQF treatment, the viability of PLC/PRF/5 and SNU449 cells was assessed using a crystal violet assay, with absorbance measured at 570 nm [18]. For the colony formation assay, 500 cells per well were treated with various HQF concentrations (0.15625, 0.3125, and 1.25 mg/mL) until colonies formed, followed by fixation and staining with crystal violet [19]. The wound healing assay involved creating a scratch on a confluent cell layer, followed by treatment with HQF (4.830 mg/mL for PLC/PRF/5 and 3.072 mg/mL for SNU449) at different time points (0, 12, 24, and 48 h), with scratch widths analyzed using Image J [20].

### Network pharmacology

To identify HQF-related targets, data was gathered from TCMSP (https://old.tcmsp-e.com/tcmsp.php), BATMAN-TCM (http://bionet.ncpsb.org.cn/batman-tcm/index.php), and SwissADME (http://www.swissadme.ch/about.php) databases [21–23]. The TCGA-LIHC dataset, sourced from the TCGA database [24], provided the basis for detecting differentially expressed genes (DEGs) between normal and HCC tissues using the Wilcoxon rank-sum test. These DEGs were subsequently considered as potential HCC targets. Kyoto Encyclopedia of Genes and Genomes (KEGG) pathway enrichment analysis was performed through the KOBAS database [25], and Gene Ontology (GO) enrichment analysis was carried out with the R package "ClusterProfiler" [26].

### RNA-seq

Liver tissues from the HQF high-dose (n = 3) and control (n = 3) groups in the Akt/Nras model were subjected to RNA-Seq analysis. RNA extraction was performed with Trizol, and 1 μg of total RNA was used to construct libraries with the TruSeq RNA Sample Prep Kit (Illumina). Sequencing was conducted on the Illumina Novaseq 6000 platform, and raw reads were cleaned using fastp. HISAT2 was used for genome alignment and FPKM quantification, while HTSeq-count provided gene read counts [27]. DEGs were identified through DESeq2 analysis, applying a threshold of adjusted *p* < 0.05 and a fold change greater than 1.2. GO, KEGG, and Gene Set Enrichment Analysis (GSEA) analyses were performed to determine significantly enriched functional pathways [28].

### HQF-mediated antiproliferative and apoptotic effects with immune cell profiling

Apoptosis was measured by identifying cells simultaneously stained with Annexin V and Propidium Iodide, following the kit instructions [29]. The assay was performed according to the protocol of manufacturer. The antiproliferative effect of HQF on PLC5/PRF/5 and SNU449 cells was evaluated using an EdU assay kit [30].

Single-cell suspensions from mouse tissues were subjected to flow cytometry. After lysing red blood cells, the cells were placed in FACS buffer and blocked with anti-mouse CD16/CD32 to prevent Fc receptor binding. Cells were then stained with fluorochrome-conjugated antibodies (CD8a, F4/80, Ly-6G, CD3, PD-1, Fixable Viability Dye, Ly-6C, CD86, CD25, CD11b, CD206, CTLA-4, FOXP3, NK1.1, CD49b, CD4) for 30 min on ice. Post-staining, cells were preserved in 1% paraformaldehyde, followed by data acquisition through flow cytometry and subsequent analysis with FlowJo software. Fixable viability dye was used to exclude non-viable cells, and specific immune cell populations were gated based on marker expression.

### Western blotting

Cells and tissues were lysed using RIPA buffer containing protease and phosphatase inhibitors. Equal amounts of protein were loaded onto SDS- Polyacrylamide Gel Electrophoresis (PAGE), with GAPDH used as a loading control. Following electrophoresis, proteins were transferred to PVDF membranes, blocked, and incubated with specific primary antibodies targeting PI3K, P-PI3K, AKT, P-AKT, mTOR, P-mTOR, cleaved caspase-3, Bax, and Bcl-2. After washing, membranes were incubated with HRP-conjugated secondary antibodies. Protein signals were detected using chemiluminescent reagents and captured with a chemiluminescence imaging system.

### Molecular docking

Molecular docking is a powerful method for identifying potential therapeutic compounds and predicting interactions between ligands and targets at the molecular level. In this study, AutoDock Tools was used to analyze the docking of seven components from HQF with PI3K, validating the findings from network pharmacology and RNA-seq analyses. Component structures were prepared in AutoDock Vina by adding hydrogen atoms, calculating charges, and identifying rotatable bonds. Protein structures were obtained from the RCSB PDB database (https://www.rcsb.org/) and processed with PyMOL to remove non-protein elements, such as bound ligands. The docking grid was set based on the coordinates of the original ligand to encompass the entire protein. Rigid docking was performed on the receptor, and the genetic algorithm was employed during the docking process. Docking results were generated using AutoGrid and AutoDock, and the lowest-energy pose was identified through clustering analysis. PyMOL was then used to produce high-quality 2D representations of interactions between small molecules and the protein, highlighting binding residues and bonds.

### Effect of HQF combined with the PD-1 inhibitor in vivo

For the combination experiment, c-Met/sgPten cells (2.5 × 10^5^ cells/mL) were subcutaneously injected into the right flank of C57BL/6 J mice. Five days after injection, when tumors had formed, the mice were randomly assigned into four groups (n = 6): control, HQF, PD-1 inhibitor, and HQF combined with PD-1 inhibitor. The control group received daily oral gavage of saline for 20 days. The HQF group was treated with daily oral gavage of HQF (33.54 g/kg/day) for 20 days. The PD-1 inhibitor group was administered an intraperitoneal injection of PD-1 inhibitor (RMP1-14, Bio X Cell, NH, USA; 100 μg/injection) every 3 days for 20 days. The combination group received daily oral gavage of HQF (33.54 g/kg/day) along with intraperitoneal injections of PD-1 inhibitor (100 μg/injection) every 3 days on the same schedule. Tumor volume and body weight were measured every 3 days. Tumor volume was calculated using the formula: V = W × L^2^/2, where W represents the shortest tumor diameter and L represents the longest tumor diameter, both measured in millimeters.

### Safety evaluation of HQF

Eighteen six-week-old male C57BL/6 J mice were selected for the HQF toxicity study. Randomly assigned, the treatment group received daily oral doses of HQF for 4 weeks, while the control group was given distilled water. At the end of the treatment, mice were sacrificed, and liver, spleen, lung, kidneys, brain, and small intestine samples were collected for histopathological analysis using HE staining [31]. Blood samples were gathered to assess liver function through the measurement of AST, ALT, ALP, γ-GT, and TBIL levels in serum [32].

### Statistical analysis

Data were derived from experiments conducted with a minimum of three replicates and analyzed using GraphPad Prism. Results are expressed as mean ± SEM. Statistical analysis involved one-way ANOVA, Student’s t-test, and Wilcoxon test, with significance defined as *p* < 0.05.

## Results

### Composition analysis of HQF

**Fig. 1:**
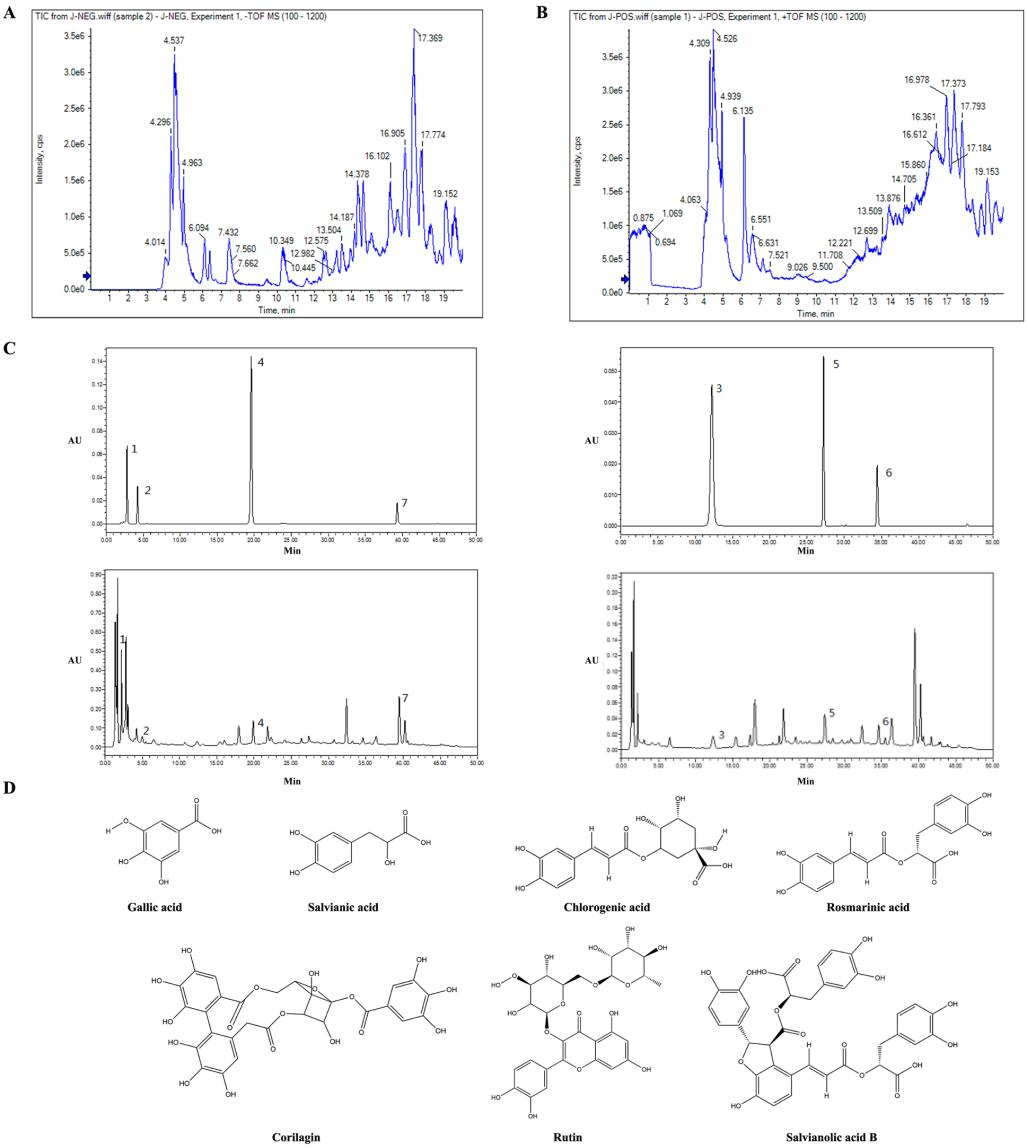
Qualitative and quantitative analysis of the chemical components in HQF was conducted using ultra-performance liquid chromatography-quadrupole time-of-flight tandem mass spectrometry (UPLC-TOF–MS/MS) and HPLC. **A**, **B** Total ion chromatogram of HQF decoction in both positive (**A**) and negative (**B**) ion modes. **C** HPLC analysis of standard compounds and HQF at 280 nm (Left) and 350 nm (Right), respectively. **D** Chemical structure of seven chemical components identified by HPLC in HQF

In this study, the composition of HQF was preliminarily identified by analyzing its retention times and fragment ion peaks. The total ion chromatograms in both positive and negative ion modes are shown in Fig. 1A and B. Based on prior studies of each herbal component in the HQF, along with chemical standards and references [33–37], twenty-nine chemical constituents were identified in negative ion mode, six in positive ion mode, and six in both ion modes. Among the 41 chemical constituents identified, phenolic acids and flavonoids were the dominant categories, each comprising 16.2% of the total constituents, followed by iridoid glycosides and organic acids, each contributing 13.5%. Detailed information on these compounds is provided in Table 1. These findings indicate that HQF is rich in phenolic acids and flavonoids.

**Table 1:** Chemical composition mass spectrometry information

No	Name	Retention	Adduct/char	Decoction	Precursor mass	Found at mass	Mass error	Score	MS/MS fragment
1	L-arginine	4.28	[M + H]^+^	C_6_H_14_N_4_O_2_	175.119	175.119	0.4	97.1	175.1168, 130.0956, 116.0714, 98.0654, 70.0661, 60.05555
	L-arginine	4.28	[M–H]^−^	C_6_H_14_N_4_O_2_	173.104	173.1038	− 3.7	100	131.0849
2	Trigonelline	4.58	[M + H]^+^	C_7_H_7_NO_2_	138.055	138.0545	− 3.3	99.7	138.0548, 110.0617, 92.0493, 78.0338, 65.0386, 53.0397
3	L-proline	4.62	[M + H]^+^	C_5_H_9_NO_2_	116.071	116.0707	0.9	98.5	116.0705, 70.0667, 68.0504
4	Quinic acid	4.63	[M–H]^−^	C_7_H_12_O_6_	191.056	191.0559	− 0.9	93.7	173.0469, 127.0393, 109.0295, 93.0353, 85.0303
5	Pipecolic acid	4.89	[M + H]^+^	C_6_H_11_NO_2_	130.086	130.0867	3.4	81.3	84.0816, 70.0657, 56.0505
6	L-malic acid	4.92	[M–H]^−^	C_4_H_6_O_5_	133.014	133.0149	4.8	97.2	133.0146, 115.0042, 72.9935, 71.0145
7	Citric acid	4.97	[M–H]^−^	C_6_H_8_O_7_	191.02	191.0207	4.8	99.2	191.0183, 173.0112, 147.0284, 129.0183, 111.0094, 87.0093, 85.0302
8	Guanosine	6.13	[M–H]^−^	C_10_H_13_N_5_O_5_	282.084	282.0841	− 0.9	99.2	282.0829, 150.0416, 133.0161, 108.0192
	Guanosine	6.13	[M + H]^+^	C_10_H_13_N_5_O_5_	284.099	284.0996	2.4	100	152.0571, 135.0303, 110.0344
9	Deacetylasperulosidic acid	6.37	[M–H]^−^	C_16_H_22_O_11_	389.109	389.1091	0.5	97	389.1096, 227.0559, 209.0453, 183.0656, 165.0558, 147.0453, 139.0402, 121.0297, 89.0246
10	Isoleucine	6.48	[M + H]^+^	C_6_H_13_NO_2_	132.102	132.1013	− 4.8	96.7	86.0960, 69.0708, 57.0777
11	Succinic acid	6.57	[M–H]^−^	C_4_H_6_O_4_	117.019	117.0193	0.1	99.3	117.0218, 99.9296, 73.0293
12	Gallic acid	7.43	[M–H]^−^	C_7_H_6_O_5_	169.014	169.0144	0.8	77.4	169.0138, 127.3365, 125.0254, 124.0167, 123.0096, 107.0136, 83.0128, 81.0349, 79.0192, 69.0350
13	Geniposidic acid	9.46	[M–H]^−^	C_16_H_22_O_10_	373.114	373.1138	− 0.5	98.3	373.1084, 211.0607, 167.0727, 149.0607, 123.0452, 89.0246
14	Salvianic acid	10.37	[M–H^]−^	C_9_H_10_O_5_	197.046	197.0456	0.4	99.4	197.0451, 179.0353, 135.0453, 123.0451, 122.0366, 109.0289, 105.0343, 89.0385, 72.9932
15	Chlorogenic acid	12.53	[M–H]^−^	C_16_H_18_O_9_	353.088	353.0881	0.8	97.9	353.0894, 191.0574, 179.0358, 135.0459, 85.0294
16	Geniposide	12.65	[M–H]^−^	C_17_H_24_O_11_, HCOOH	449.13	449.1305	1.1	72.4	449.1352, 403.1255, 241.0724, 209.0454, 139.0397, 101.0242
17	Loganic acid	13.00	[M–H]^−^	C_16_H_24_O_10_	375.13	375.1295	− 0.5	91.5	375.1313, 331.0933, 213.0763, 169.0889, 113.0244, 95.0495, 69.0348
	Loganic acid	13.00	[M + H]^+^	C_16_H_24_O_10_	377.144	377.1452	2.5	98.9	377.1469, 359.1325, 243.0882, 198.0666, 172.0872, 145.0763, 69.0337
18	Esculin	13.32	[M–H]^−^	C_15_H_16_O_9_	339.072	339.0721	− 0.2	74	339.0714, 259.1225, 177.0197, 133.0289, 105.0351
19	Asperuloside	13.53	[M + H]^+^	C_18_H_22_O_11_	415.123	415.1236	0.3	93.9	235.0601, 193.0497, 175.0381, 147.0434, 119.0499, 91.0532
20	Corilagin	14.66	[M–H]^−^	C_27_H_22_O_18_	633.073	633.0754	3.3	99.3	633.0783, 463.0637, 301.0004, 275.0209
21	3,4-dihydroxybenzaldehyde	14.87	[M–H]^−^	C_7_H_6_O_3_	137.024	137.0245	0.3	99.3	137.0246, 136.0178, 119.0268, 108.0229, 91.0214, 81.0390, 65.0052
22	Eleutheroside E	15.25	[M–H]^−^	C_34_H_46_O_18_, HCOOH	787.267	787.2701	4.4	100	787.3577, 741.2537, 579.2080, 417.1560, 181.0529
23	Esculetin	15.53	[M–H]^−^	C_9_H_6_O_4_	177.019	177.0195	0.9	95.8	177.0195, 149.0239, 133.0285, 121.0318, 107.0139, 105.0340, 93.0337, 89.0387, 77.0386, 65.0401
24	Rutin	16.02	[M–H]^−^	C_27_H_30_O_16_	609.146	609.1478	2.8	96.3	609.1510, 300.0287, 271.0232
	Rutin	16.02	[M + H]^+^	C_27_H_30_O_16_	611.161	611.161	0.6	97.2	465.1036, 303.0515, 129.0551, 85.0284
25	Calycosin-7-O-*β*-D-glucoside	16.38	[M–H]^−^	C_22_H_22_O_10_, HCOOH	491.119	491.12	1	100	491.1283, 283.0615, 268.0388, 239.0360, 211.0427, 195.0455
26	Isoquercitrin	16.40	[M–H]^−^	C_21_H_20_O_12_	463.088	463.0886	0.9	97.5	463.0888, 300.0264, 271.0247, 255.0303, 151.0045
27	Kaempferol-3-O-rutinoside	16.46	[M–H]^−^	C_27_H_30_O_15_	593.151	593.1532	3.3	98.1	593.1545, 285.0381, 256.0356, 227.0368
28	Ellagic acid	16.50	[M–H]^−^	C_14_H_6_O_8_	300.999	300.9995	1.6	94.5	301.0018, 229.0159, 173.0250, 145.0298
29	Cistanoside D	16.54	[M–H]^−^	C_31_H_40_O_15_, HCOOH	697.235	697.2384	5	79.5	697.2778, 651.2333, 375.1362, 339.1259, 191.0577, 136.0185, 151.0412
30	3,4-dicaffeoylquinic acid	16.68	[M–H]^−^	C_25_H_24_O_12_	515.119	515.1204	1.7	92.4	515.1203, 353.0880, 335.0758, 191.0573, 179.0336, 173.0456, 135.0439
31	Hesperetin	16.93	[M + H]^+^	C_16_H_14_O_6_	303.086	303.0865	0.6	91.9	303.0882, 177.0554, 153.0196, 145.0285, 117.0337, 67.0181
32	P-coumaric acid	16.96	[M–H]^−^	C_9_H_8_O_3_	163.04	163.0401	0.4	96.9	119.0509, 93.0350, 65.0403
33	Rosmarinic acid	17.35	[M–H]^−^	C_18_H_16_O_8_	359.077	359.0775	0.7	99.5	197.0451, 179.0353, 135.0453, 123.0451, 122.0366, 109.0289, 105.0343, 89.0385, 72.9932
34	Salvianolic acid B	17.38	[M–H]^−^	C_36_H_30_O_16_	717.146	717.149	4.1	99.7	717.1565, 519.0935, 339.0541, 321.0432, 295.0630, 185.0258
35	Ononin	17.70	[M–H]^−^	C_22_H_22_O_9_, HCOOH	475.125	475.1246	0.1	100	300.0204, 267.0668, 252.0455
36	Salvianolic acid A	17.78	[M–H]^−^	C_26_H_22_O_10_	493.114	493.1144	0.8	95.7	433.1132, 313.0725, 295.0631, 203.0350, 197.0458, 185.0529, 159.0452, 135.0456, 109.0303
37	Isomucronulatol 7-O-glucoside	18.15	[M–H]^−^	C_23_H_28_O_10_	463.161	463.161	0.1	95.4	301.1089, 286.0884, 271.0602, 164.0487, 135.0440, 121.0309, 89.0239
38	Quercetin	18.63	[M–H]^−^	C_15_H_10_O_7_	301.035	301.0352	− 0.7	93.9	273.0393, 245.0519, 211.0407, 183.1041, 179.0003, 151.0044, 121.0293, 107.0143, 65.0035
	Quercetin	18.63	[M + H]^+^	C_15_H_10_O_7_	303.05	303.0509	3.3	92.2	303.0510, 274.0497, 257.0440, 229.0488, 201.0535, 165.0194, 153.0175, 137.0220, 81.0335
39	Wogonin	18.75	[M–H]^−^	C_16_H_12_O_5_	283.061	283.0615	0.9	88.5	283.0626, 268.0396, 239.0358, 223.0393, 211.0406, 195.0454, 183.0455, 148.0165, 135.0093
	Wogonin	18.75	[M + H]^+^	C_16_H_12_O_5_	285.076	285.076	0.8	97.9	285.0780, 270.0543, 253.0509, 225.0560, 213.0562, 158.0727, 141.0669, 137.0239, 115.0541, 89.0388
40	Tectorigenin	18.76	[M–H]^−^	C_16_H_12_O_6_	299.056	299.0563	0.8	75.7	299.0563, 284.0330, 255.0313, 227.0366, 211.0410, 199.0403, 183.0447, 148.0172, 108.0223
41	Naringenin	19.46	[M–H]^−^	C_15_H_12_O_5_	271.061	271.0611	− 0.4	94.1	271.0595, 190.9940, 177.0154, 151.0043, 119.0497, 107.0134, 83.0155, 65.0022

### Quantification of seven compounds in HQF via HPLC

Based on the results above, the contents of seven compounds were quantified, and the HPLC chromatogram is shown in Fig. 1C, with compound structures presented in Fig. 1D. At different wavelengths and retention times, various compounds were detected. At 280 nm, salvianolic acid B showed the highest content (0.303%), followed by danshensu (0.213%), gallic acid (0.271%), and corilagin (0.063%). At 350 nm, rosmarinic acid had the highest content (0.222%), followed by rutin (0.054%) and chlorogenic acid (0.034%) (Table 2).

**Table 2:** HPLC analysis of HQF

NO	Components	Content (mg/g)	Regression equation	R^2^	Linearity range (μg/mL)
1	Gallic acid	2.710	Y = 2000000X-3885.3	0.9996	0.0257–0.514
2	Salvianic acid	2.132	Y = 639179X-2521.3	0.9990	0.052–1.04
3	Chlorogenic acid	0.337	Y = 1000000X-37251	0.9995	0.109–2.187
4	Corilagin	0.626	Y = 20000000X-31323	0.9998	0.12625–2.525
5	Rutin	0.535	Y = 1000000X-12370	0.9998	0.0453–0.907
6	Rosmarinic acid	2.220	Y = 133139X-8367.1	0.9999	0.192–3.833
7	Salvianolic acid B	3.032	Y = 923342X-9652.3	1.0000	0.026175–0.5235

### HQF inhibited the c-Met/sgPten-induced HCC subcutaneous tumor

**Fig. 2:**
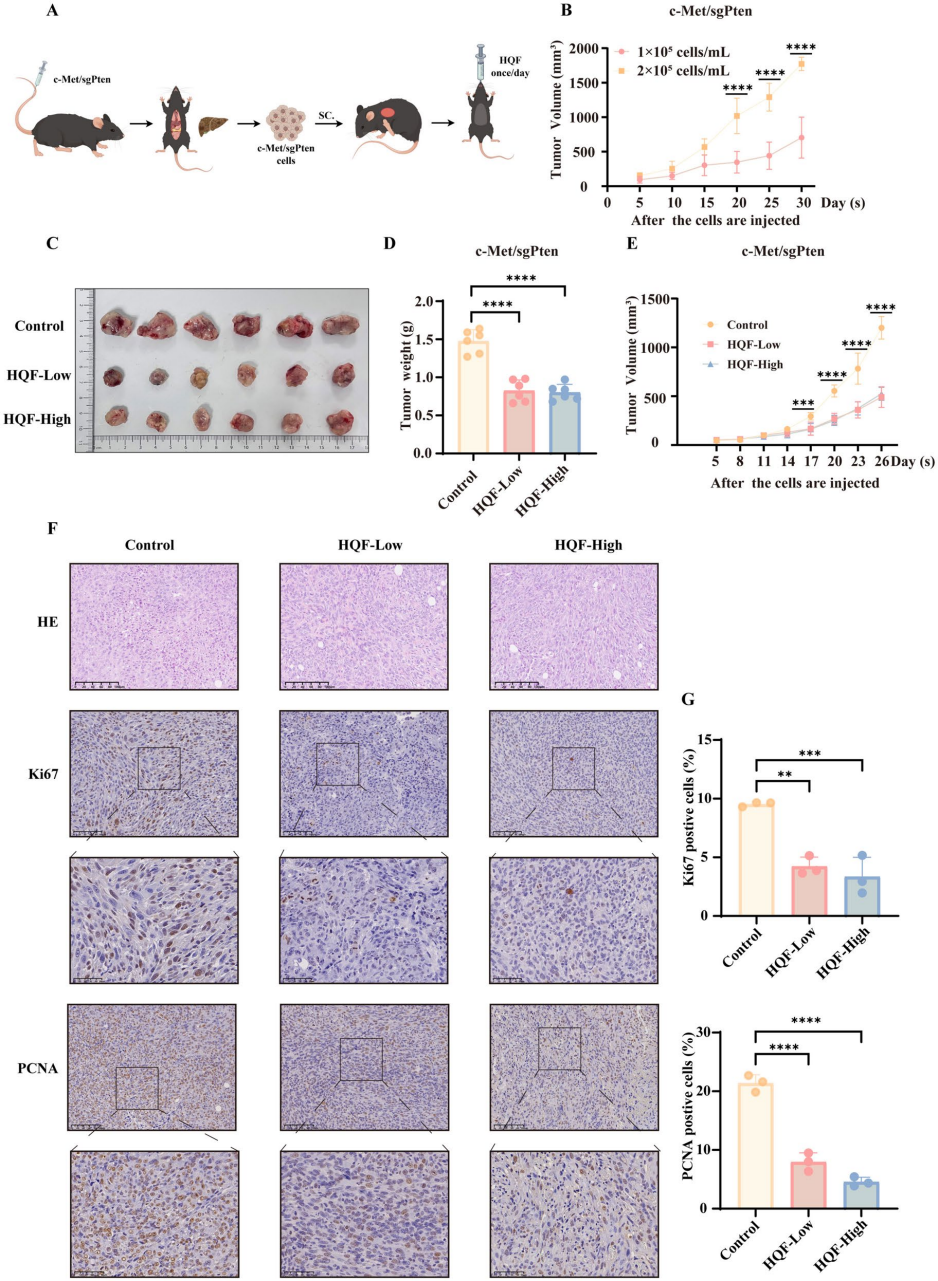
HQF demonstrated anti-HCC efficacy in subcutaneous tumor-bearing mice. **A** A schematic diagram depicting the establishment of the c-Met/sgPten model and treatment of mice with HQF. **B** The relationship between c-Met/sgPten cell seeding density and tumor size changes. **C** HQF reduced tumor size. **D**, **E** Tumor weight (**D**) and tumor volume (**E**) changes were monitored in mice treated with vehicle or HQF. **F** HQF improved liver pathology and inhibited the expression of Ki-67 and PCNA. **G** Quantitative analysis of immunohistochemistry. Significance was indicated as **p < 0.01, ***p < 0.001, or ****p < 0.0001

To evaluate the effects of HQF on subcutaneous tumors induced by c-Met/sgPten, we initially established an HCC model through hydrodynamic injection of the c-Met/sgPten plasmid. After isolating HCC cells from the tumor tissues, we carefully selected the optimal cell concentration and used these cells to establish a subcutaneous tumor model (Fig. 2A). As shown in Fig. 2B, the subcutaneous tumor volume in the high-density cell group gradually increased from day 20 and displayed a significant difference compared to the low-density cell group by the end of the experiment (*p* < 0.0001). Following oral administration of HQF, both HQF-treated groups exhibited a significant reduction in tumor volume and weight. On day 22, the tumor weight in the control group and HQF-high group was 1.47 g and 0.80 g, respectively (*p* < 0.0001, Fig. 2C, D), while the tumor volume was 1200.93 mm^3^ in the control group and 492.01 mm^3^ in the HQF-high group (*p* < 0.0001, Fig. 2E).

In the HCC subcutaneous tumor tissues, HE staining typically revealed features such as abnormal cellular structure, increased nuclear-to-cytoplasmic ratio, cellular pleomorphism, irregular hyperchromatic nuclei, as well as areas of necrosis and stromal invasion. These pathological characteristics provided a foundation for evaluating the pharmacodynamic effects of therapeutic interventions, as changes in these features can indicate the efficacy of the treatment. After HQF treatment, improvements such as restoration of cellular architecture, reduction in the nuclear-to-cytoplasmic ratio, decreased cellular pleomorphism, reduced irregular hyperchromatic nuclei, and alleviation of necrotic areas and stromal invasion should be observed, further supporting the therapeutic efficacy of HQF (Fig. 2F). Ki67 and PCNA are important markers for assessing the malignancy of HCC and are commonly measured through immunohistochemistry. Compared with control group, HQF significantly reduced the expression of Ki67 and PCNA in the subcutaneous tumor tissues (Fig. 2F, *p* < 0.01).

### HQF inhibited the Akt/Nras-induced HCC orthotopic tumor

**Fig. 3:**
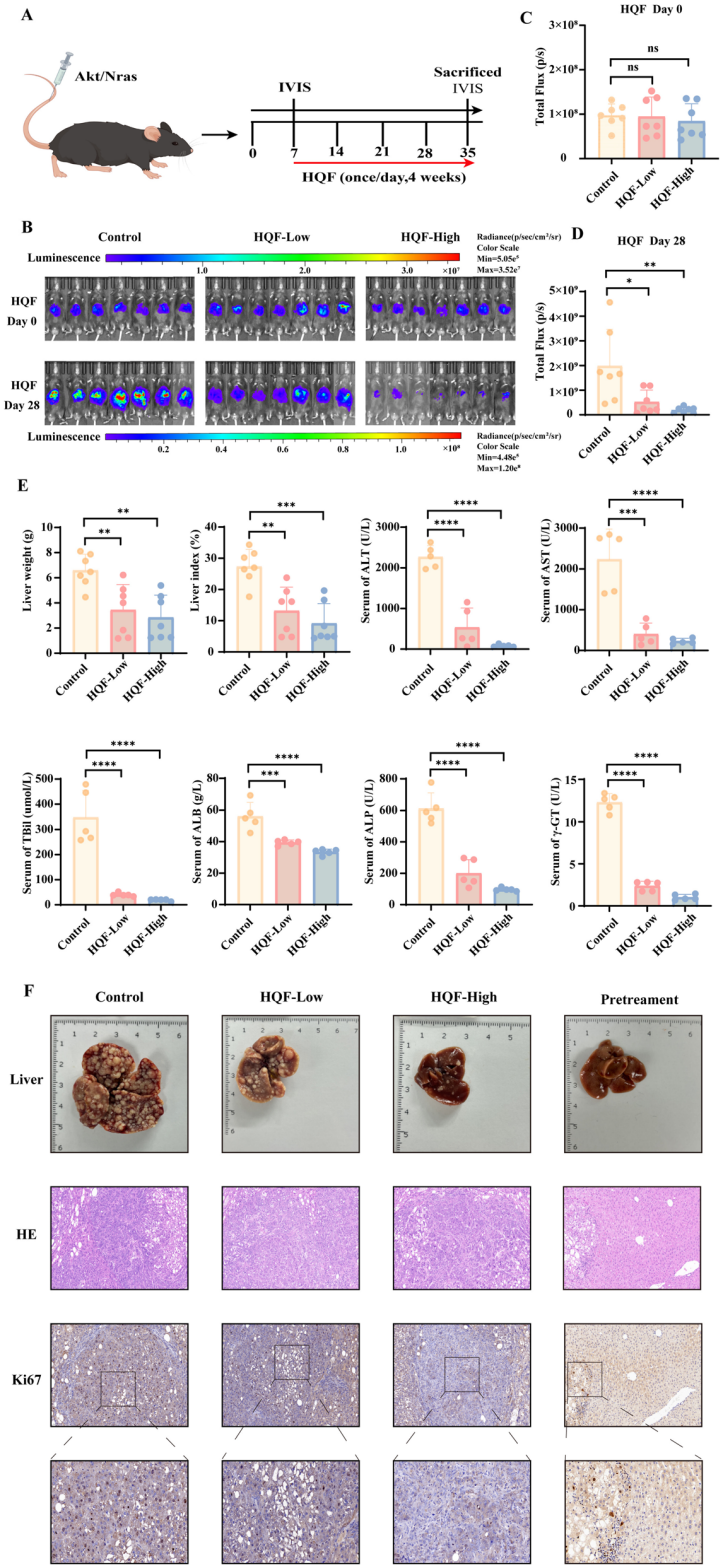
HQF demonstrated anti-HCC efficacy in an orthotopic liver cancer model. **A** A schematic diagram illustrating the establishment of the Akt/Nras model and the treatment of mice with HQF. **B** Bioluminescence imaging showed the efficacy of vehicle and HQF (low and high dose) in mice bearing Akt/Nras-Luc orthotopic tumors. **C**–**D** Quantification of fluorescence intensity was conducted for in vivo imaging of mice treated with vehicle or HQF (low and high dose). **E** HQF reduced liver weight, liver index, ALT, AST, TBIL, ALB, ALP, and γ-GT levels. **F** HQF improved liver pathology and inhibited the expression of the tumor proliferation marker Ki67. Significance was indicated as *p < 0.05, **p < 0.01, ***p < 0.001, or ****p < 0.0001

After demonstrating the inhibitory effect of HQF on the subcutaneous tumor model, we extended the study to the Akt/Nras-induced orthotopic model to further validate its therapeutic potential in a more clinically relevant setting (Fig. 3A). To further assess the therapeutic impact of HQF in the orthotopic HCC model, in vivo imaging system (IVIS) imaging was employed to monitor tumor progression before and after treatment. 7 days after hydrodynamic injection of the Akt/Nras plasmid, no significant difference in total flux was observed between the low- and high-dose HQF groups compared to the control group (Fig. 3B, C). After 28 days of HQF treatment, the total flux in both HQF groups demonstrated a dose-dependent reduction compared to the control group (Fig. 3D, *p* < 0.05).

Additionally, to investigate the effects of HQF on liver injury in Akt/Nras-induced HCC mice, liver weight, liver index, and liver injury markers were measured. As shown in Fig. 3E, liver weight and liver index were reduced in the model group compared to the control group (*p* < 0.01). By evaluating liver injury markers, the potential protective effects of HQF on liver damage during HCC progression were further assessed. Additionally, compared to the control group, the HQF group showed improvements in ALT, ALB, AST, ALP, TBIL, and γ-GT levels (Fig. 3E, *p* < 0.01). Encouragingly, HQF reduced the Akt/Nras-induced morphological changes in a dose-dependent manner and was able to inhibit the expression of the Ki-67 marker (Fig. 3F and Fig. S1).

### HQF inhibited proliferation and migration of HCC cells

**Fig. 4:**
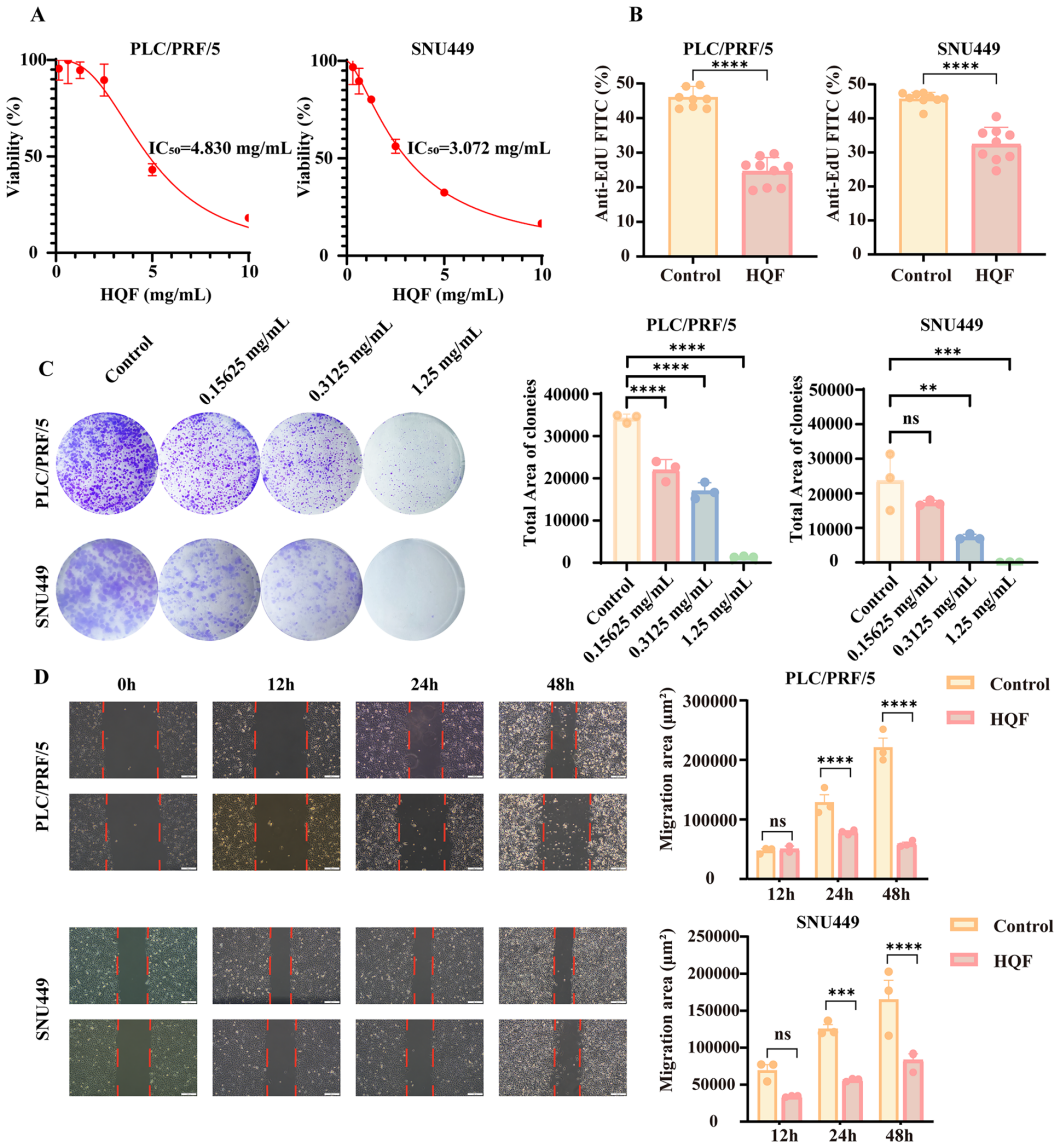
HQF inhibited the growth of HCC cells in vitro. **A** Inhibitory effects of HQF on HCC cell lines were quantified using Crystal Violet staining after 48 h of treatment. **B** Anti-proliferative capacity of PLC/PRF/5 and SNU449 HCC cell lines following HQF treatment was assessed by EdU staining. **C** Colony formation assay of PLC/PRF/5 and SNU449 HCC cell lines after HQF treatment. Representative images (left panel) and quantification (right panel) were shown. **D** Wound healing assay was performed to assess the migration ability of PLC/PRF/5 and SNU449 cells at 0, 12, 24, and 48 h. Representative images (left panel) and quantification (right panel) were shown. Significance was indicated as **p < 0.01, ***p < 0.001, or ****p < 0.0001

To evaluate the inhibitory effect of HQF on HCC cells, HCC cell lines were treated with specific concentrations of HQF for 48 h. The results showed that HQF significantly suppressed HCC cell viability in a dose-dependent manner (Fig. 4A). After 48 h of treatment, the IC_50_ values for PLC/PRF/5 and SNU449 cells were 4.830 mg/mL and 3.072 mg/mL, respectively (Fig. 4A).

To further confirm the antiproliferative effect of HQF, we performed the EdU incorporation assay. HQF treatment significantly reduced the number of EdU-positive cells compared with the control group (Fig. 4B, *p* < 0.0001), indicating that HQF effectively inhibits the proliferation of HCC cells. In the colony formation assay, HQF-treated groups displayed a significant reduction in colony-forming ability (Fig. 4C, *p* < 0.01), further supporting its antiproliferative effect. In the wound healing assay, HQF significantly delayed wound closure compared with the control group (Fig. 4D, *p* < 0.001), suggesting that HQF suppresses the migratory ability of HCC cells. Collectively, these results demonstrate that HQF inhibits the proliferation, colony formation, and migration of HCC cells, highlighting its potential therapeutic value in impeding HCC progression.

### Network pharmacology of HQF in the treatment of HCC

**Fig. 5:**
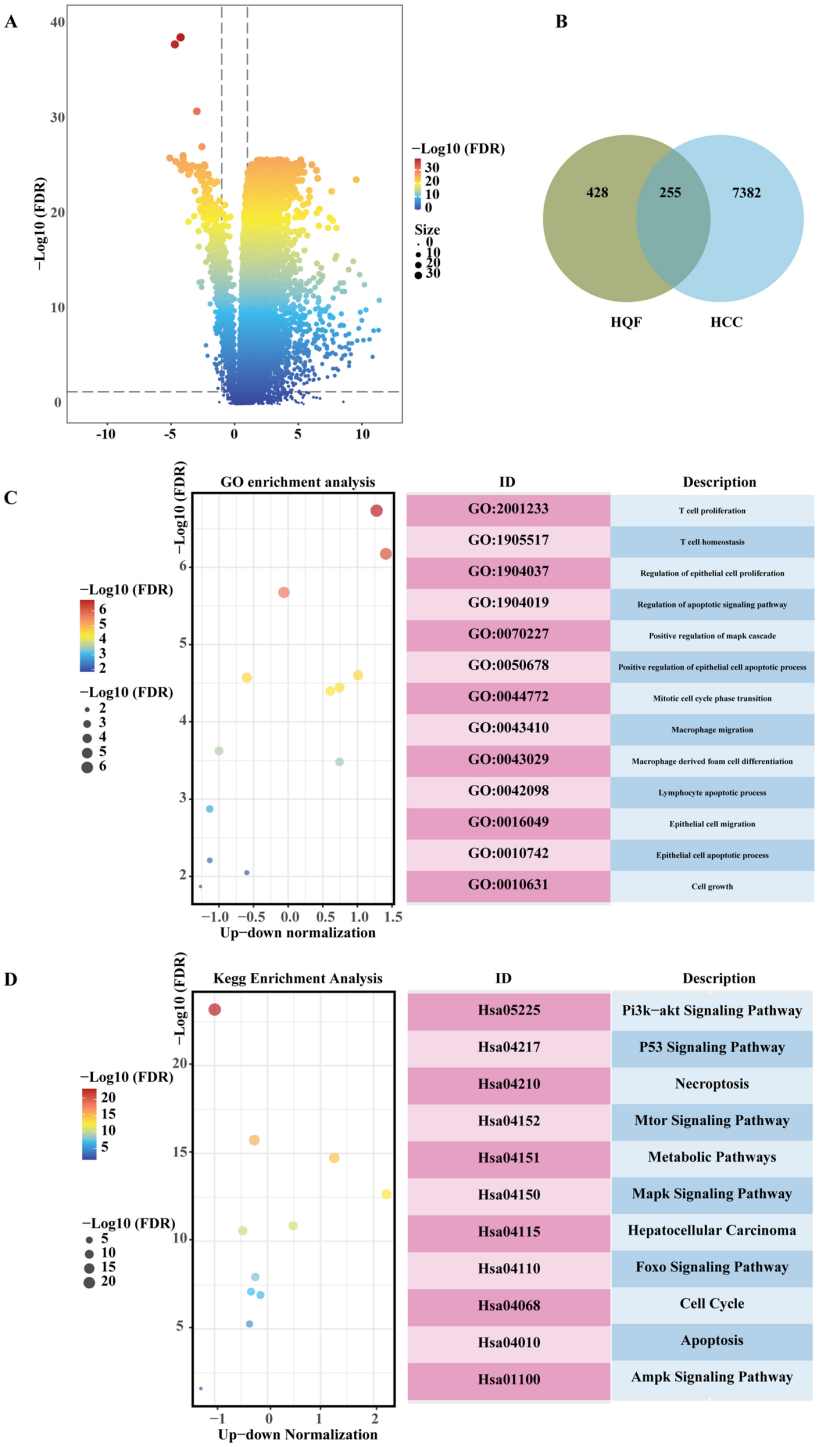
Network pharmacology analysis of HQF in the treatment of HCC. **A** Differential expression analysis between normal and HCC patients in the TCGA-LIHC dataset. **B** The overlapped targets between HQF and HCC. **C**, **D** GO (**C**) and KEGG (**D**) analysis of the overlapped targets

To further explore the potential mechanisms of HQF in treating HCC, network pharmacology analysis was conducted to identify key signaling pathways and targets, providing a more comprehensive understanding of the mechanism of action of HQF. First, 7637 DEGs were identified between the normal group and the HCC group in the TCGA-LIHC cohort (Fig. 5A). Then, based on the chemical components identified by the LC-TOF–MS analysis, 783 potential targets of HQF were collected from the TCMSP, SwissADME, and BAN-TCM databases (Fig. 5B). A total of 255 overlapping targets mapped to HQF-related compounds and HCC-related genes were identified (Fig. 5B). GO enrichment analysis indicated involvement in T cell proliferation, heterogeneity, and epithelial cell proliferation (Fig. 5C). KEGG pathway analysis identified several key signaling pathways associated with HCC, including the PI3K-AKT signaling pathway, p53 signaling pathway, apoptosis pathway, mTOR signaling pathway, metabolic pathways, MAPK signaling pathway, FOXO signaling pathway, cell cycle, and AMPK signaling pathway. Although each of these pathways plays a critical role in HCC development and progression, the PI3K/AKT/mTOR signaling pathway and apoptosis pathway emerged as particularly significant for further investigation. The PI3K/AKT/mTOR pathway, as a primary regulator of cell growth and survival, has been shown to promote tumor cell proliferation, inhibit apoptosis, and enhance drug resistance when aberrantly activated factors that are crucial in HCC progression (Fig. 5D).

### RNA-seq analysis of HQF in the treatment of HCC

**Fig. 6:**
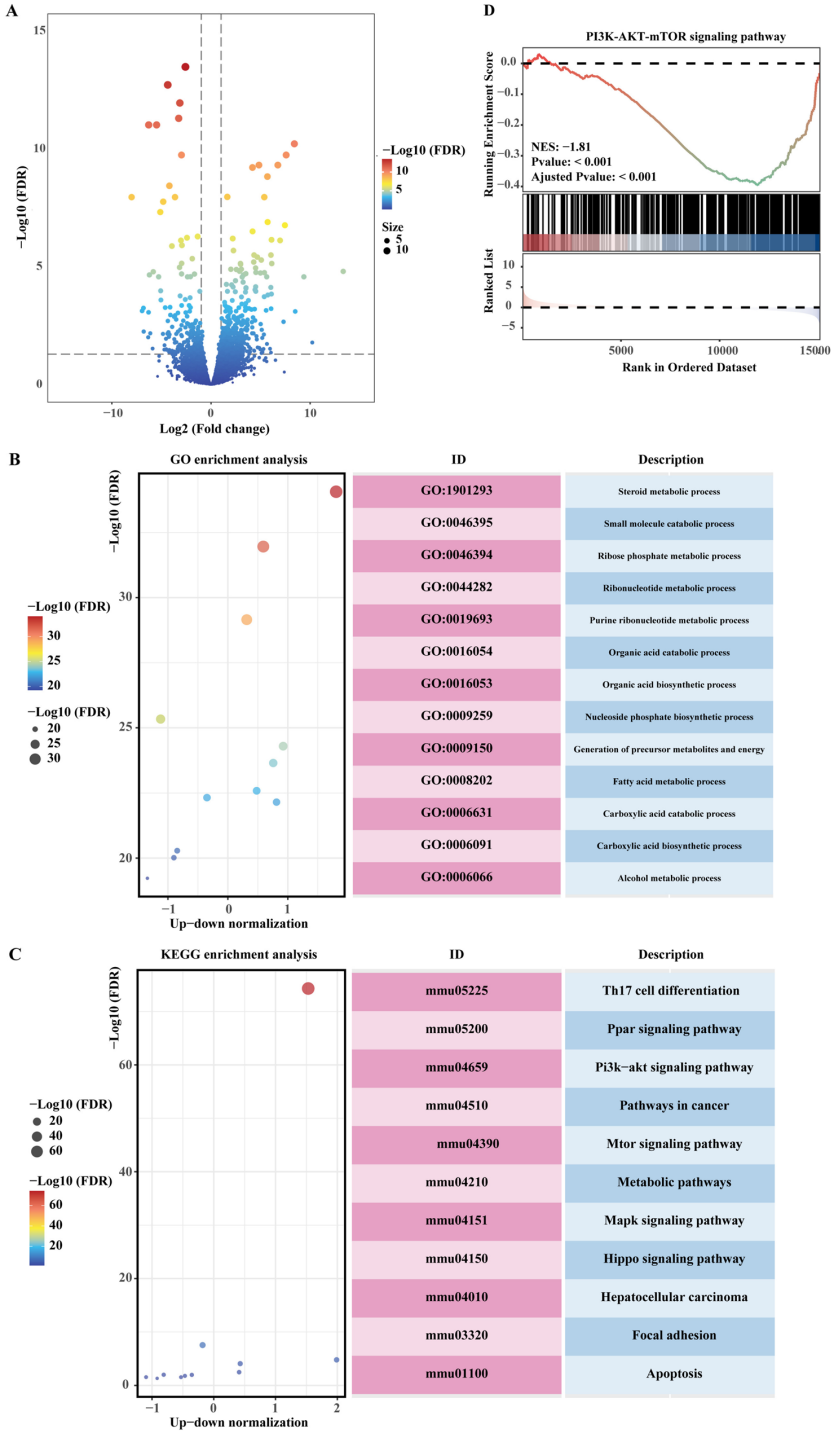
RNA-seq and pathway enrichment analysis in HCC cells treated with HQF or vehicle. **A** Differential expression analysis between vehicle- and HQF-treated HCC cells. **B**, **C** GO (**B**) and KEGG (**C**) analysis of DEGs. **D** GSEA enrichment analysis of the PI3K/AKT/mTOR signaling pathway

Following network pharmacology analysis, RNA-seq was conducted to validate the predicted key targets and pathways. RNA-seq analysis revealed 457 upregulated genes and 249 downregulated genes in the HQF treatment group (Fig. 6A). GO enrichment analysis indicated associations with steroid and small molecule metabolism (Fig. 6B), while KEGG pathway analysis identified multiple enriched pathways, including Th17 cell differentiation, PPAR signaling pathway, PI3K-AKT signaling pathway, cancer-related pathways, mTOR signaling pathway, metabolic pathways, MAPK signaling pathway, HIPPO signaling pathway, HCC, focal adhesion, and apoptosis signaling pathway (Fig. 6C). Additionally, GSEA further confirmed significant downregulation of these pathways in the HQF treatment group (Fig. 6D). By comparing the gene expression profiles of HCC tissues between the HQF treatment and control groups, we assessed the differential expression of pathways and genes consistent with the network pharmacology findings. This analysis highlighted the PI3K/AKT/mTOR and apoptosis pathways, verifying the molecular mechanisms of HQF in HCC.

### HQF promoted apoptosis in HCC via inhibiting PI3K/AKT/mTOR pathway

**Fig. 7:**
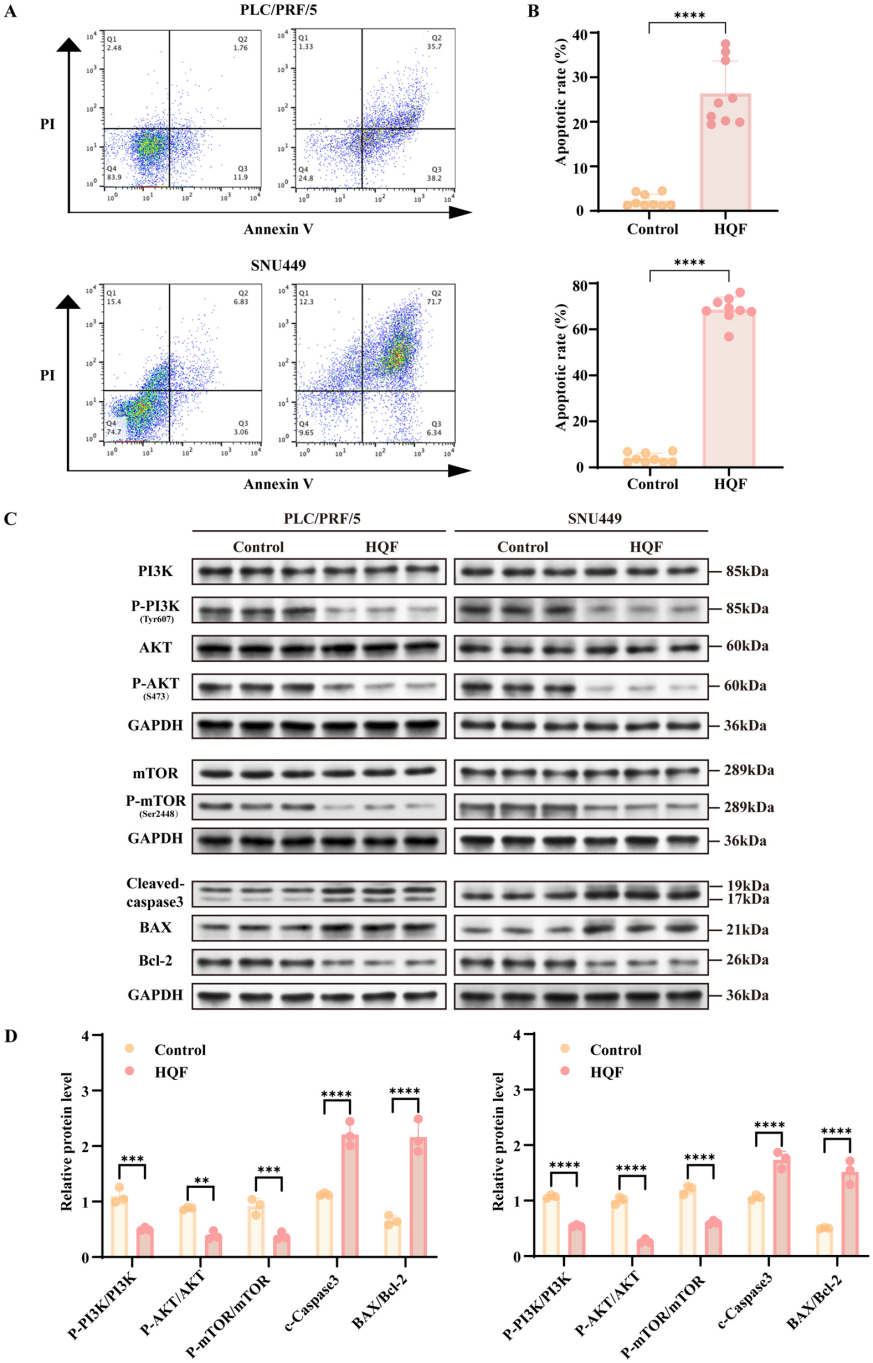
Evaluation of the effects of HQF on apoptosis and key pathway proteins in HCC cell lines. **A**, **B** Analysis of apoptosis (**A**) and its quantitative assessment (**B**) by flow cytometry. **C**, **D** Validation of the PI3K/AKT/mTOR pathway and apoptosis-related proteins (Bax, Bcl-2, Cleaved Caspase-3). **C** shows the protein bands, and (**D**) represents the quantitative analysis of protein expression. Significance was indicated as **p < 0.01, ***p < 0.001, or ****p < 0.0001

**Fig. 8:**
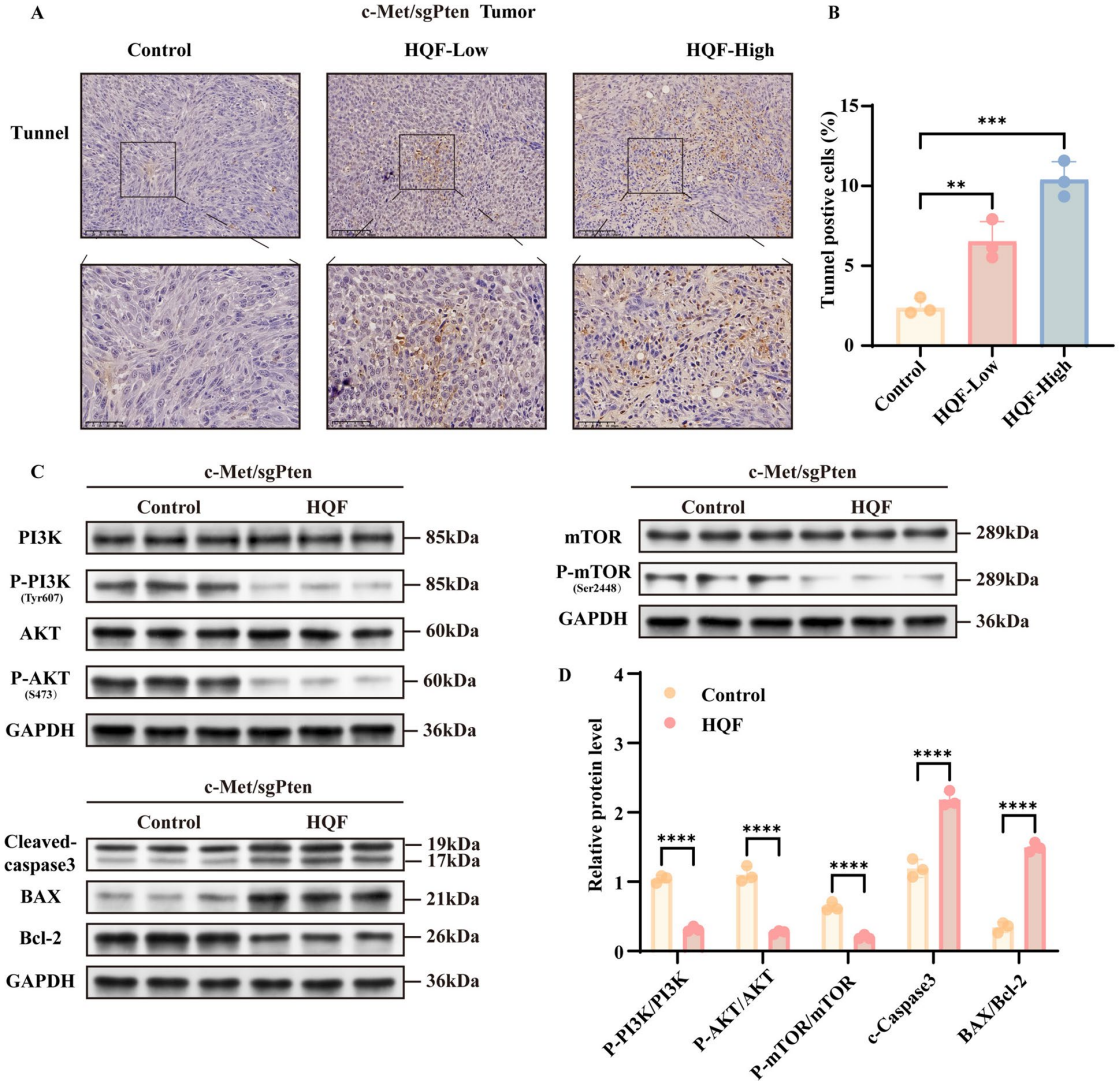
Effects of HQF on apoptosis and protein regulation in the c-Met/sgPten animal model. **A**, **B** The positive expression rates of TUNEL in the c-Met/sgPten model were analyzed by immunohistochemical staining (magnification, 20 × and 40 ×) (**A**) and quantitatively assessed (**B**). **C**, **D** Regulation of apoptosis-related proteins via the PI3K/AKT/mTOR pathway, with (**C**) representing the protein bands and (**D**) showing the quantitative analysis of protein expression. Significance was indicated as **p < 0.01, ***p < 0.001, or ****p < 0.0001

Based on the findings from network pharmacology and RNA-seq analysis, HQF treatment for HCC was found to be closely related to apoptosis and the PI3K/AKT/mTOR pathway. We further performed experimental validation to confirm these key mechanisms. As shown in Fig. 7A, B, HQF significantly promoted apoptosis in SNU449 and PLC/PRF/5 cell lines compared to the control group (Fig. 7A, B  *p* < 0.0001). Mechanistically, both high- and low-dose HQF groups showed an increasing trend in the levels of pro-apoptotic proteins Bax/Bcl_2_ and cleaved caspase-3, while components of the PI3K/AKT/mTOR signaling pathway, including PI3K, P-PI3K (Tyr607), AKT, P-AKT (S473), mTOR, and P-mTOR (Ser2448), were downregulated compared to the control group (Fig. 7C, D, *p* < 0.01). Furthermore, in the c-Met/sgPten animal model, TUNEL staining confirmed a significant increase in apoptotic cells (Fig. 8A, B, *p* < 0.01). Compared to the control group, both high- and low-dose HQF groups showed an upward trend in the levels of pro-apoptotic proteins Bax/Bcl_2_ and cleaved caspase-3, while components of the PI3K/AKT/mTOR pathway, including PI3K, P-PI3K (Tyr607), AKT, P-AKT (S473), mTOR, and P-mTOR (Ser2448), were downregulated (Fig. 8C, D, *p* < 0.0001). These findings indicate that HQF promotes apoptosis in HCC by inhibiting the PI3K/AKT/mTOR pathway.

### PI3K targeted molecular docking analysis of seven key active compounds

**Fig. 9:**
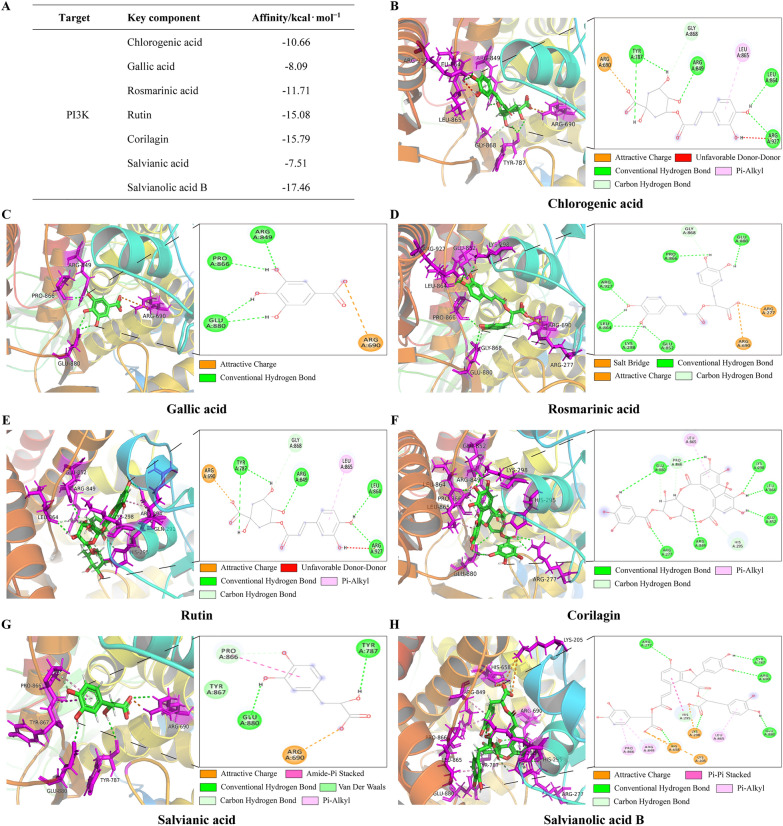
Molecular docking results. (**A**) Binding energy table of key components with PI3K. **B**–**H** Molecular docking of PI3K with chlorogenic acid, gallic acid, rosmarinic acid, rutin, corilagin, salvianic acid, and salvianolic acid B. Note: In the docking images, the stick model represents the active molecule; the green stick structure denotes the carbon backbone of small molecules, red dots indicate oxygen atoms, white dots represent hydrogen atoms, and dotted lines show chemical bonds between the active compound and amino acid residues

Key target PI3K was docked with seven active compounds: chlorogenic acid, gallic acid, rosmarinic acid, rutin, corilagin, salvianic acid, and salvianolic acid B (Fig. 9A). Generally, a docking score with an absolute value > 4.25 indicates moderate activity, > 5.0 signifies good binding affinity, and > 7.0 represents strong binding affinity. Based on the docking results, the top three “target protein-compound” conformations were PI3K with salvianolic acid B, PI3K with corilagin, and PI3K with rutin. Visualization was conducted using PyMOL software (Fig. 9B–H).

### HQF modulated T cell proportions in the immune microenvironment of HCC mice

**Fig. 10:**
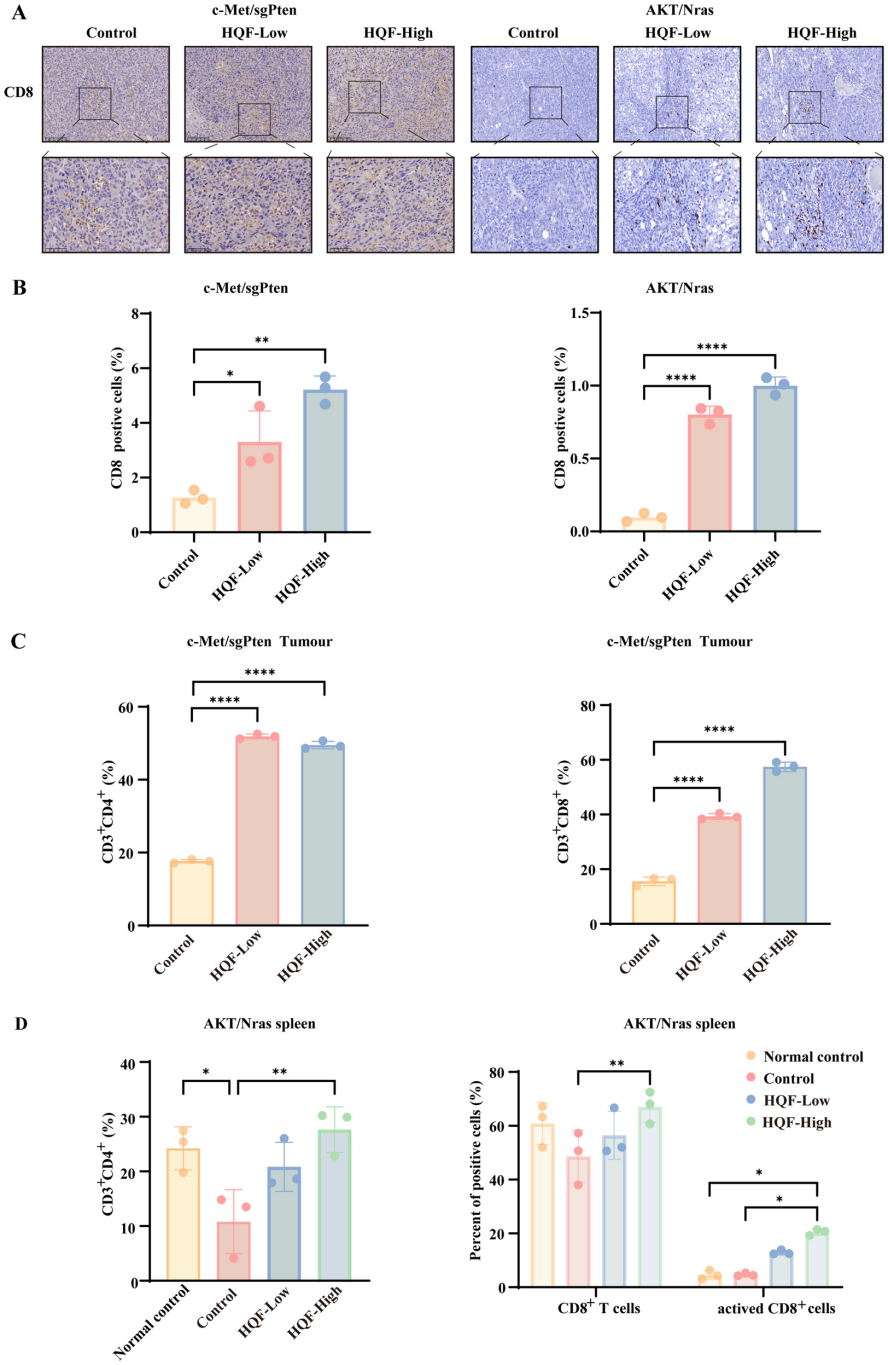
HQF altered the proportion of T cells in the immune microenvironment of HCC mice. **A**, **B** Positive expression rates of CD8 (**A**) and their quantitative analysis (**B**) were assessed by immunohistochemical staining (magnification, 20 × and 40 ×) in the c-Met/sgPten and Akt/Nras models. **C**, **D** The positive expression rates of CD4 and CD8 were analyzed by flow cytometry. **C** shows the positive expression rates of CD4 and CD8 in subcutaneous tumors from the c-Met/sgPten model, while (**D**) shows the positive expression rates of CD4, CD8, and activated CD8 in the spleens from the Akt/Nras model. Significance was indicated as *p < 0.05, **p < 0.01, or ****p < 0.0001

T cells play a crucial role in the HCC immune microenvironment. HQF significantly altered T cell proportions in HCC mice, enhancing immune response modulation. First, immunohistochemical analysis was conducted to observe the localization and infiltration of CD8^+^ T cells in the tumor microenvironment, providing a spatial understanding of the immune response. As shown in Fig. 10A, B, CD8^+^ T cell infiltration in tumors was significantly increased in both the c-Met/sgPten-induced subcutaneous tumor model and the Akt/Nras-induced orthotopic model compared to the control group (*p* < 0.05). Flow cytometry was then performed to further quantify and validate these findings by assessing the proportions of CD3^+^CD4^+^ and CD3^+^CD8^+^ T cells in the c-Met/sgPten and Akt/Nras models, allowing for a more detailed evaluation of systemic immune activation. The results revealed that, compared to the control group, higher proportions of CD3^+^CD4^+^ and CD3^+^CD8^+^ T cells were observed following HQF treatment (Fig. 10C, D and Fig. S2-S3, *p* < 0.05).

### HQF combined with PD-1 significantly enhances anti-HCC effects

**Fig. 11:**
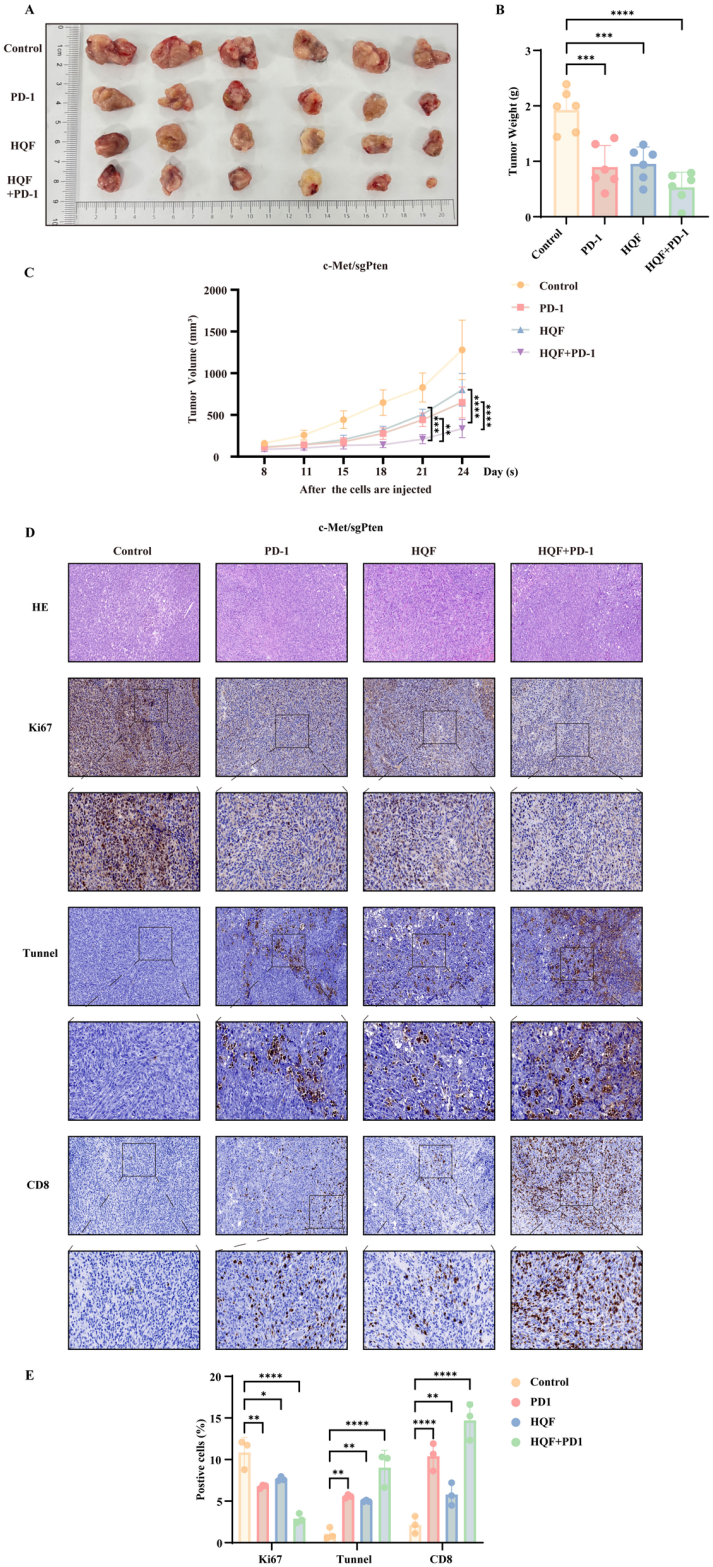
The efficacy of HQF in synergizing with PD-1 to reverse HCC. (A-C) HQF and PD-1 combination therapy reduced tumor size (**A**), weight (**B**), and volume (**C**). **D** HQF and PD-1 improved liver pathology, inhibited the levels of Ki-67, and promoted the levels of apoptosis cells, enhanced the CD8^+^ cell area. **E** The quantitative analysis of Ki-67, apoptosis cells, and CD8^+^ cell. Significance was indicated as *p < 0.05, **p < 0.01, ***p < 0.001, or ****p < 0.0001

Previous findings suggested that HQF treatment for HCC may be associated with an increase in CD8^+^ T cell infiltration. Therefore, we hypothesized that combining HQF with PD-1 would result in improved therapeutic outcomes. To test this hypothesis, we conducted experiments using the c-Met/sgPten model. Excitingly, compared to the HQF-only treatment group, the combination of HQF and PD-1 significantly reduced tumor size, volume, and weight (Fig. 11A–C, p < 0.001). Additionally, Ki-67 expression was significantly decreased, and the proportion of apoptotic cells was markedly increased. More importantly, after combined treatment with HQF and PD-1, the proportion of CD8^+^ T cells was further elevated compared to the HQF-only group (Fig. 11D, E, p < 0.01).

### Safety evaluation

**Fig. 12:**
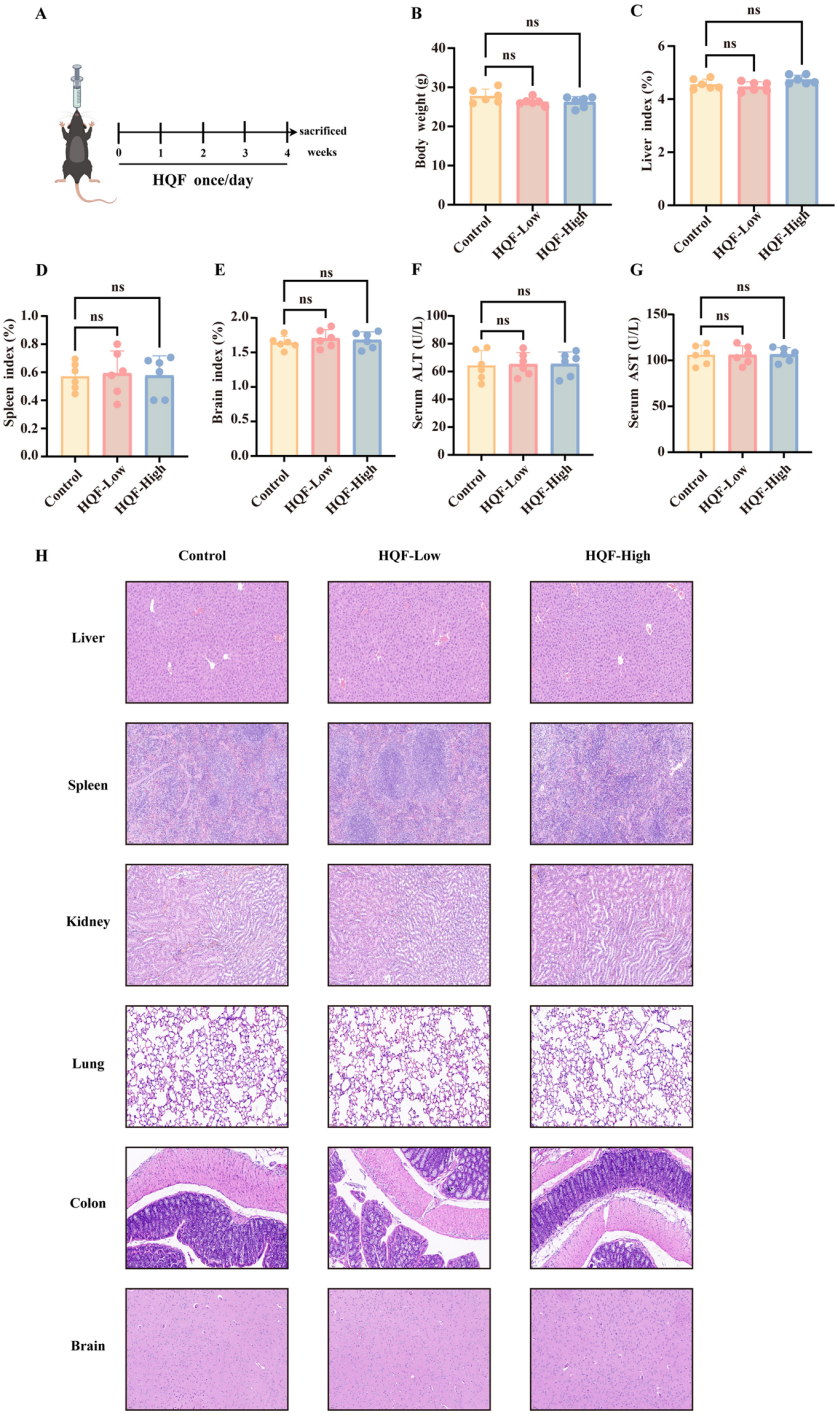
HQF exhibited no significant host toxicity in vivo. **A** A schematic diagram of the HQF toxicity assessment. **B**–**G** Body weight, liver index, spleen index, brain index, ALT, and AST changes were monitored in mice receiving vehicle or HQF treatment. **F** Histological examination of major organs (liver, spleen, lung, kidneys, brain, and colon) in normal mice after treatment with vehicle or HQF (low or high dose)

Although HQF is effective in treating liver diseases, its organ toxicity remains underexplored. To ensure clinical safety, we conducted organ toxicity experiments in mice (Fig. 12A). No significant differences were observed in body weight, organ indices (liver, spleen, and brain), ALT and AST levels between the HQF-treated and control groups (Fig. 12B–G). Additionally, HE staining revealed no pathological damage in the liver, spleen, kidneys, lung, colon, and brain (Fig. 12H). These findings suggested that HQF exhibited no observable organ toxicity in mice, supporting its potential safety for clinical use.

## Discussion

With its growing impact on global mortality, cancer remains a serious public health concern. Recently, tumor immunotherapy has taken center stage as a transformative approach in cancer therapies and drug innovation [38]. The distinctive feature of TCM in cancer treatment lies in its ability to target both tumor cells and regulate immune cells, achieving a dual-modulation effect. While the clinical effectiveness of HQF in treating HCC has been documented in prior research, the mechanisms underlying its effects remain largely unexplored. This study aimed to investigate the anti-tumor mechanisms of HQF and its regulatory effects on the immune microenvironment, thereby highlighting its dual therapeutic action in HCC.

To this end, we employed two innovative tumor models in this study: a subcutaneous model constructed using primary HCC cells (c-Met/sgPten) and an orthotopic model established via hydrodynamic injection of Akt/Nras/SB plasmids into the tail vein. These models provide reliable platforms for evaluating tumor growth and immune regulation, and they better recapitulate the genetic and microenvironmental characteristics of HCC compared to traditional models. By utilizing these advanced models, this study elucidates the anti-tumor mechanisms of HQF and its regulatory effects on the immune microenvironment, further highlighting its dual therapeutic potential in the treatment of HCC.

During HCC treatment, HQF functions by suppressing tumor cell proliferation, initiating apoptosis, and causing cell cycle interruption at critical stages [39]. Our observations of SNU449 and PLC/PRF/5 cells morphology following HQF treatment showed reduced cell density, membrane shrinkage, and the presence of apoptotic bodies. These preliminary findings demonstrated that HQF suppressed the proliferation of SNU449 and PLC/PRF/5 cells while also enhancing apoptotic activity. Through further evaluation using TUNEL and flow cytometry, we found that HQF induces apoptosis in HCC cells. Our study revealed that HQF enhances apoptosis by increasing Bax/Bcl_2_ expression and activating cleaved caspase-3. This implied that HQF could act through the PD-1/PD-L1 axis, laying the groundwork for exploring its potential to boost the effectiveness of PD-1-based therapies. HQF is composed of six herbs: *V. coloratum*, *A. membranaceus*, *S. miltiorrhiza*, *C. phaeocaulis*, *O. diffusa*, and *P. urinaria* L. Cui et al. discovered that *O. diffusa* injection inhibits HepG2 cell growth and triggers apoptosis, potentially involving the regulation of the Bcl-2 signaling pathway [40]. *P. urinaria* downregulates Bcl-2 expression in BEL-7404 cells, thereby disrupting cell survival mechanisms and promoting apoptosis in HCC [41]. This observation was consistent with our study, which shows that HQF can downregulate Bcl-2 expression in HCC cells. Our research on HQF not only corroborated these well-established roles but also revealed that HQF treatment significantly modulated the expression of these proteins. Specifically, HCC cells treated with HQF showed reduced Bcl-2 expression and elevated levels of Bax and Caspase-3, suggesting that HQF induces apoptosis via the mitochondrial pathway.

Previously, the complete chemical profile of HQF had not been fully identified. Using HPLC and LC-TOF–MS, we identified seven compounds in HQF, including chlorogenic acid, gallic acid, rosmarinic acid, rutin, corilagin, salvianic acid, and salvianolic acid B, providing valuable insights for further clinical studies. GO analysis indicated that these seven chemical components may regulate tumor cell proliferation, production, and apoptosis through various metabolic processes. KEGG and Western blot analyses pointed to the involvement of the PI3K/AKT/mTOR pathway in mediating the observed biological effects. By interacting with the PI3K/AKT/mTOR pathway, salvianolic acid B, rutin, and chlorogenic acid have been shown to provide therapeutic benefits in managing diabetes, breast cancer, and esophageal cancer [42–44]. These compounds may work by hindering PI3K activation, limiting AKT phosphorylation, and subsequently dampening mTOR signaling.

Molecular docking results with PI3K indicated that salvianolic acid B, corilagin, and rutin may play significant roles in the anti-HCC effects of HQF. As a primary active constituent of *S. miltiorrhiza*, salvianolic acid B has demonstrated anti-tumor potential in various studies.Salvianolic acid B elevates p-MST1 and p-YAP, blocking cell proliferation and migration driven by TGF-β_1_ and MST1/2 inhibitor XMU-MP-1 [45]. Research on an H22 HCC mouse model highlighted the robust anti-tumor potential of salvianolic acid B. The findings revealed that salvianolic acid B not only curbed tumor growth but also mitigated inflammation-related immune activity by regulating the TLR4/MyD88/NF-κB pathway [46]. Additionally, salvianolic acid B modulated the gut microbiota of tumor-bearing mice, bringing their microbial community structure closer to that of the normal group, further enhancing its anti-tumor effects. These observations revealed that salvianolic acid B may regulate tumor growth and immune responses through various signaling pathways, highlighting its therapeutic potential. By targeting essential proteins, corilagin activated both mitochondrial and receptor-mediated apoptotic pathways, effectively inducing cell death in HCC cells. By disrupting mitochondrial membrane potential, it triggered the release of cytochrome c. This, in turn, activated caspase-9 and caspase-3, leading to the cleavage of PARP [47]. Rutin exerted its chemopreventive effects against HCC by reducing liver and tumor marker enzymes, normalizing membrane-bound enzymes and electrolytes, and mitigating the damage induced by N-Nitrosodiethylamine and phenobarbital, thereby restoring liver function and inhibiting tumor progression in male Wistar rats [48]. Overall, the chemical constituents of HQF identified by HPLC have demonstrated potential to inhibit HCC progression through multiple pathways. The presence of these compounds in HQF, along with their strong therapeutic associations, underscored HQF’s multi-target mechanisms in HCC treatment.

The PI3K/AKT/mTOR pathway plays a critical role in tumor cell survival, proliferation, and immune evasion, often contributing to the creation of an immunosuppressive microenvironment that undermines the efficacy of PD-1 therapy [49]. In this study, we found that HQF significantly induces apoptosis and inhibits tumor growth in HCC cells by suppressing the PI3K/AKT/mTOR pathway. Furthermore, HQF treatment enhanced T-cell infiltration in the tumor microenvironment and improved immune activity. These findings suggest that HQF can simultaneously regulate tumor cells and the immune microenvironment, resulting in dual inhibition of tumor immune evasion and proliferation.

The inhibition of the PI3K/AKT/mTOR pathway not only restores apoptotic signaling in tumor cells but may also promote the release of apoptosis-related signaling molecules, such as DAMPs and HMGB1, and pro-inflammatory cytokines, such as IL-12 and IFN-γ, which activate anti-tumor immune responses. Studies have shown that these factors can attract effector T cells into the tumor microenvironment and enhance their activation, thereby improving immune-mediated tumor killing [50, 51]. In addition, HQF may further support T-cell anti-tumor activity by modulating the proportion of immunosuppressive cell populations in the tumor microenvironment, such as MDSCs and Tregs. Previous studies have demonstrated that the inhibition of the PI3K/AKT/mTOR pathway can reduce the numbers of MDSCs and Tregs and decrease their secretion of immunosuppressive factors, such as TGF-β and IL-10, thereby improving immune activity within the tumor microenvironment [52, 53]. These effects align with our findings of increased T-cell infiltration and functional activation following HQF treatment. These results suggest that HQF may initiate a positive feedback mechanism through the suppression of the PI3K/AKT/mTOR pathway, where enhanced tumor cell apoptosis promotes T-cell activation, and activated T cells further improve immune-mediated tumor killing in the tumor microenvironment. Nevertheless, further studies are required to elucidate the specific roles of key factors involved in these mechanisms and to understand how HQF balances its dual effects on tumor cells and T cells. Such investigations will provide a more comprehensive theoretical basis for the potential application of HQF in HCC therapy.

## Conclusion

This study highlights the anti-HCC potential of HQF, demonstrating its regulation of the PI3K/AKT/mTOR pathway and the T-cell immune microenvironment. These findings suggest HQF as a promising therapeutic candidate for liver cancer and provide a foundation for its future clinical application, including its potential use in combination therapies for HCC.

## Supplementary Information

Supplementary Material 1.

## Data Availability

The public data utilized in this study can be accessed through the specified websites, while additional data may be obtained from the corresponding author upon reasonable request.

## Abbreviations

AKT: Protein kinase B
ALB: Albumin
ALP: Alkaline phosphatase
ALT: Alanine aminotransferase
AST: Aspartate aminotransferase
DEGs: Differentially expressed genes
DMEM: Dulbecco’s modified eagle medium
FBS: Fetal bovine serum
GO: Gene ontology
GSEA: Gene set enrichment analysis
HCC: Hepatocellular carcinoma
HE: Hematoxylin and eosin
HPLC: High-performance liquid chromatography
HQF: Huqi formula
IVIS: In vivo imaging system
KEGG: Kyoto encyclopedia of genes and genomes
LC-TOF–MS: Liquid chromatography-time of flight-mass spectrometry
mTOR: Mammalian target of rapamycin
PAGE: Polyacrylamide gel electrophoresis
PD-1: Programmed death-1
PD-L1: Programmed death-ligand 1
PI3K: Phosphoinositide 3-kinase
SEM: Standard error of the mean
TBIL: Total bilirubin
TCM: Traditional Chinese medicine
γ-GT: Gamma-glutamyl transferase
