# Supplementary material for: Huqi formula suppresses hepatocellular carcinoma growth by modulating the PI3K/AKT/mTOR pathway and promoting T cell infiltration
Source: Chin Med. 2025 Feb 19;20:25. doi: 10.1186/s13020-025-01061-w (PMC11837637; doi:10.1186/s13020-025-01061-w)
Supplement: Supplementary file 1 — Supplementary Material 1. [file 13020_2025_1061_MOESM1_ESM.docx]

Huqi formula suppresses hepatocellular carcinoma growth by modulating the PI3K/AKT/mTOR pathway and promoting T cell infiltration

Donghao Yin^a,1^, Xiang Li^b,1^, Xuemeng Yang^a,b,1^, Xiaofei Shang^c^, Zhen Li^b,d^, Jiahao Geng^b^, Yanyu Xu^b^, Zijing Xu^b^, Zixuan Wang^b^, Zimeng Shang^e^, Zhiyun Yang^e^, Linlan Hu^b^, Quanwei Li^a^, Jiabo Wang^b,^*, Xinhua Song^b,^ *, Xiuhui Li^a,^*, Xiaojun Wang ^a,^*

^a^*Beijing YouAn Hospital, Capital Medical University, Beijing, 100069, China.*

^b^*Department of Natural Medicines*, *School of Traditional Chinese Medicine, Laboratory for Clinical Medicine, Capital Medical University, Beijing, 100069, China.*

*^c^Key Laboratory of New Animal Drug Project, Gansu Province, Key Laboratory of Veterinary Pharmaceutical Development of Ministry of Agriculture, Lanzhou Institute of Husbandry and Pharmaceutical Sciences, Chinese Academy of Agricultural Sciences, Lanzhou 730050, China.*

*^d^Cao xian People's Hospital,Shandong,* *274400, China.*

*^e^Center for Integrative Medicine, Beijing Ditan Hospital, Capital Medical University, No. 8 Jingshun East Street, Beijing 100015, People’s Republic of China.*

* Corresponding author. Beijing YouAn Hospital, Capital Medical University, Beijing, 100069, China

*E-mail* address: w_xiaojun@ccmu.edu.cn (X.W.)

^1^ These authors have contributed equally to this work.


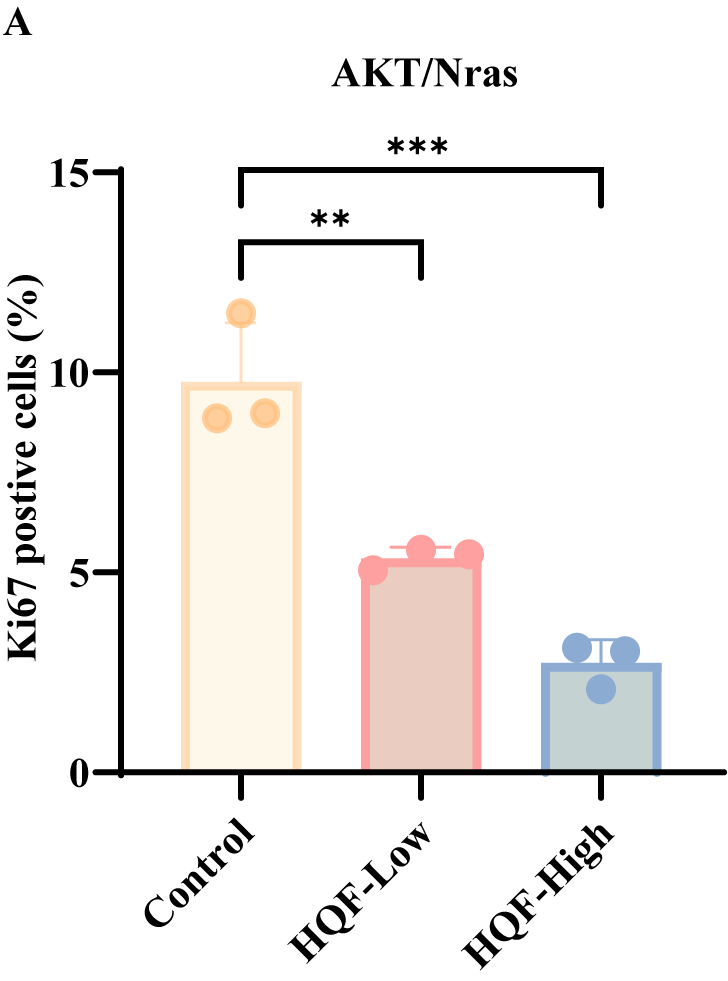


**Fig. S1.** HQF inhibited the expression of Ki-67 in the Akt/Nras model. Significance was indicated as ***p* < 0.01, or ****p* < 0.001.


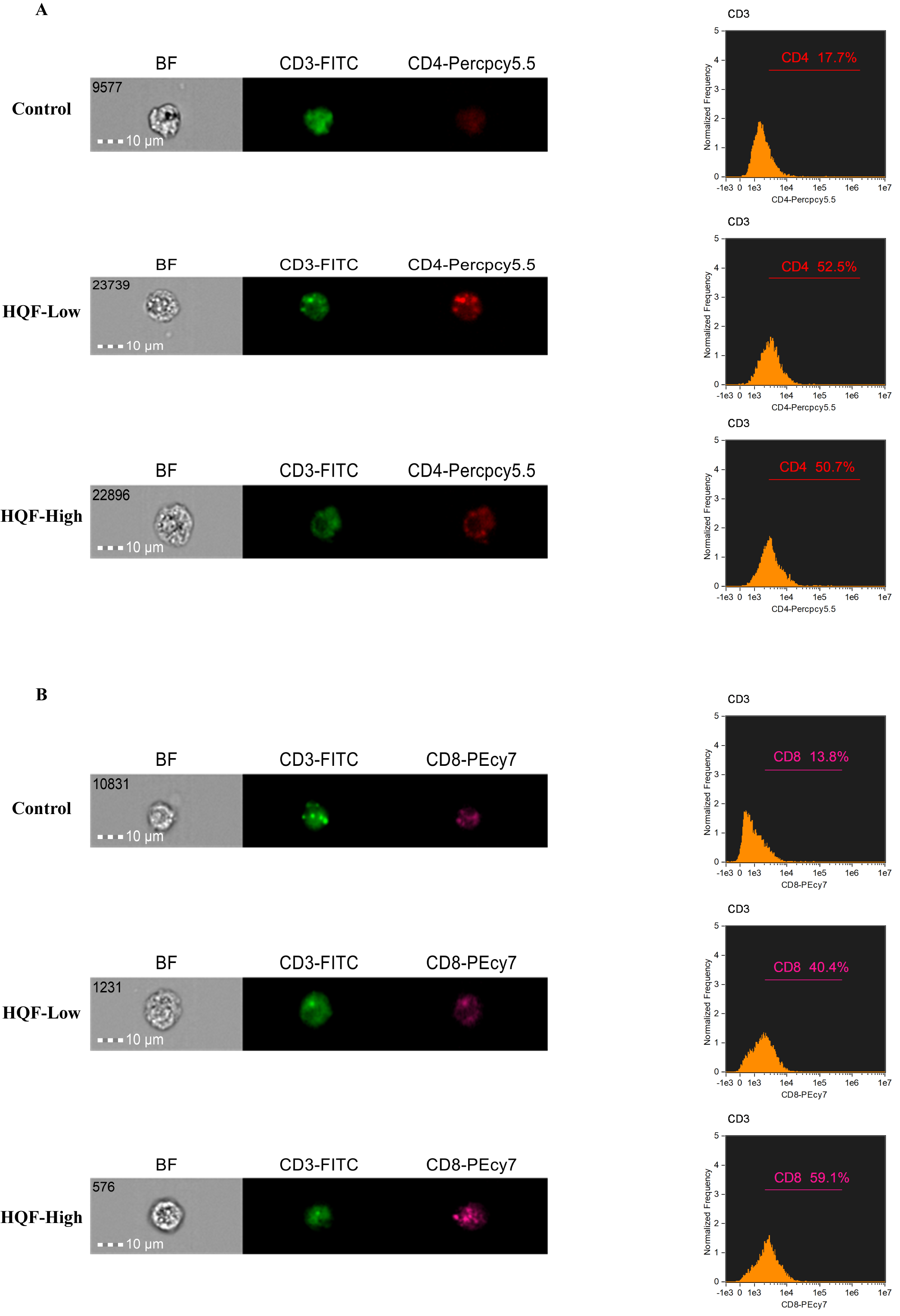


**Fig. S2.** Detection of the positive expression rates of CD4 and CD8 in subcutaneous tumors from the c-Met/sgPten model using imaging flow cytometry after HQF treatment.


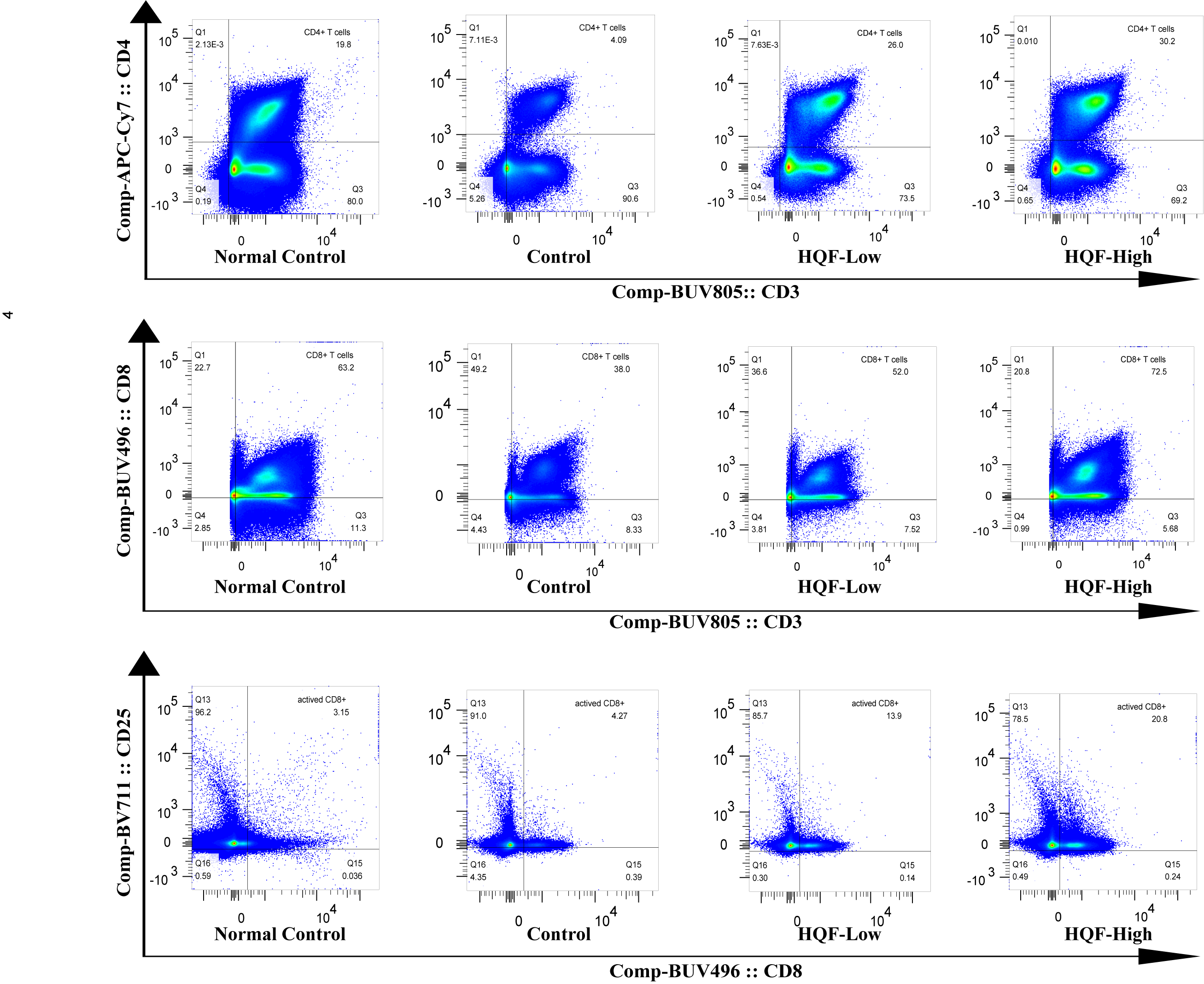


**Fig. S3.** Detection of the positive expression rates of CD4, CD8, and activated CD8 in the spleens from the Akt/Nras model after HQF treatment using flow cytometry.
